# A Sequential Multiple Assignment Randomized Trial (SMART) study of medication and CBT sequencing in the treatment of pediatric anxiety disorders

**DOI:** 10.1186/s12888-021-03314-y

**Published:** 2021-06-30

**Authors:** Bradley S. Peterson, Amy E. West, John R. Weisz, Wendy J. Mack, Michele D. Kipke, Robert L. Findling, Brian S. Mittman, Ravi Bansal, Steven Piantadosi, Glenn Takata, Corinna Koebnick, Ceth Ashen, Christopher Snowdy, Marie Poulsen, Bhavana Kumar Arora, Courtney M. Allem, Marisa Perez, Stephanie N. Marcy, Bradley O. Hudson, Stephanie H. Chan, Robin Weersing

**Affiliations:** 1 Children’s Hospital Los Angeles, Los Angeles, CA USA; 2 Department of Psychiatry, Keck School of Medicine at The University of Southern California, Los Angeles, USA; 3 Department of Pediatrics, Keck School of Medicine at the University of Southern California, Los Angeles, USA; 4 Department of Psychology, Harvard University, Cambridge, USA; 5 Department of Preventive Medicine, Keck School of Medicine at The University of Southern California, Los Angeles, USA; 6 Virginia Commonwealth University, Richmond, USA; 7 Department of Research & Evaluation, Kaiser Permanente, Los Angeles, USA; 8 Brigham And Women’s Hospital, Harvard Medical School, Boston, USA; 9 Children’s Bureau of Southern California, Los Angeles, USA; 10 Hathaway-Sycamores Child and Family Services, Altadena, USA; 11 LifeStance Health California, Encinitas, USA; 12 SDSU-UC San Diego Joint Doctoral Program in Clinical Psychology, San Diego State University, San Diego, USA

## Abstract

**Background:**

Treatment of a child who has an anxiety disorder usually begins with the question of which treatment to start first, medication or psychotherapy. Both have strong empirical support, but few studies have compared their effectiveness head-to-head, and none has investigated what to do if the treatment tried first isn’t working well—whether to optimize the treatment already begun or to add the other treatment.

**Methods:**

This is a single-blind Sequential Multiple Assignment Randomized Trial (SMART) of 24 weeks duration with two levels of randomization, one in each of two 12-week stages. In Stage 1, children will be randomized to fluoxetine or Coping Cat Cognitive Behavioral Therapy (CBT). In Stage 2, remitters will continue maintenance-level therapy with the single-modality treatment received in Stage 1. Non-remitters during the first 12 weeks of treatment will be randomized to either [1] optimization of their Stage 1 treatment, or [2] optimization of Stage 1 treatment and addition of the other intervention. After the 24-week trial, we will follow participants during open, naturalistic treatment to assess the durability of study treatment effects. Patients, 8–17 years of age who are diagnosed with an anxiety disorder, will be recruited and treated within 9 large clinical sites throughout greater Los Angeles. They will be predominantly underserved, ethnic minorities. The primary outcome measure will be the self-report score on the 41-item youth SCARED (Screen for Child Anxiety Related Disorders). An intent-to-treat analysis will compare youth randomized to fluoxetine first versus those randomized to CBT first (“Main Effect 1”). Then, among Stage 1 non-remitters, we will compare non-remitters randomized to optimization of their Stage 1 monotherapy versus non-remitters randomized to combination treatment (“Main Effect 2”). The interaction of these main effects will assess whether one of the 4 treatment sequences (CBT➔CBT; CBT➔med; med➔med; med➔CBT) in non-remitters is significantly better or worse than predicted from main effects alone.

**Discussion:**

Findings from this SMART study will identify treatment sequences that optimize outcomes in ethnically diverse pediatric patients from underserved low- and middle-income households who have anxiety disorders.

**Trial registration:**

This protocol, version 1.0, was registered in ClinicalTrials.gov on February 17, 2021 with Identifier: NCT04760275.

## Background

Anxiety disorders are highly prevalent in children, adolescents, and young adults. Approximately 12.3% of children meet formal diagnostic criteria for an anxiety disorder by age 12, and an additional 11% meet criteria by age 18, most commonly social anxiety, separation anxiety, and generalized anxiety disorders [1]. Most adult anxiety disorders begin in childhood or adolescence [2–4], suggesting that early intervention may reduce anxiety prevalence in adults, attenuate the frequent worsening of symptoms and lessen the associated impairment over time [1, 5, 6]. Early treatment of pediatric anxiety disorders may also mitigate the development or functional impact of common comorbid disorders [7, 8], including depression [1], Attention-Deficit/Hyperactivity Disorder (ADHD) [9], oppositional defiant or conduct disorder [10], substance abuse [5, 11, 12], and suicide attempts [13, 14]. More broadly, anxiety disorders confer considerable risk for lifetime impairments in overall quality of life, interpersonal relationships, physical health, finances, and academic and occupational functioning [1, 6]. They are strongly associated with years of life lost and lived in disability, especially when beginning in childhood [15].

Systematic reviews and meta-analyses have consistently shown that pediatric anxiety disorders, like their adult counterparts, are chronic, recurrent, and unstable in their diagnostic classification over time, with new anxiety disorders appearing together with or replacing the initial, primary diagnosis [16, 17], suggesting strongly that clinical trials should consider multiple anxiety disorders and aggregate symptom outcome measures. Systematic reviews and meta-analyses have also documented substantial therapeutic effects for both psychotherapy, particularly CBT (Cognitive Behavioral Therapy) [18, 19], and medication, especially SSRIs (selective serotonin reuptake inhibitors) [20–22]. While response rates to acute treatment near 60% for both CBT [23] and SSRIs [21, 22] may seem encouraging, these rates also mean that approximately 40% fail to respond. Remission rates are lower, 40–50% for both CBT [24] and SSRIs [21, 22], and even poorer in real-world, community settings (20–40%) [25–27]. Long-term follow-up in pediatric studies is rare, but data suggest that outcomes are poor and relapses common, similar to what is observed in adult anxiety disorders, where relapse approaches 60% [16, 28]. Even after gold-standard treatments in the CAMS study (Child/Adolescent Anxiety Multimodal Study) – the largest and most rigorous combined medication and CBT trial thus far – 30% were chronic non-responders and an additional 50% relapsed at least once in 4 years, despite receiving post-trial treatment [29]. Racial/ethnic minorities in the CAMS study had significantly lower remission rates in all treatment arms [30]. Long-term outcomes did not vary according to initial treatment with CBT, medication, or their combination. Predictors of poor acute treatment outcomes include more severe symptoms, more functional impairment [29, 31], low socioeconomic status (SES) [32], and a primary diagnosis of social phobia [31, 32]. More residual symptoms and functional impairment following acute treatment predict relapse, suggesting that treatment should aim to achieve remission from all anxiety disorders, and with as few residual symptoms of any kind of anxiety as possible [29]. These predictors of relapse have informed our requirements for, and definition of, clinical remission during this trial.

### Gaps in evidence this study will fill

Although many studies have separately evaluated the efficacy and effectiveness of medication and CBT in the treatment of pediatric anxiety disorders, very few have compared the effectiveness of CBT and medication head-to-head [33] (the CAMS study did, but it was an efficacy study conducted in academic centers rather than in “real world” community clinics [10]). No studies have assessed whether it is preferable to begin with CBT and then add medication if needed, or begin with medication and then add CBT if needed. Our preliminary survey of clinicians, patients, and parents indicates that treatment of essentially every child with anxiety disorder begins with the vexing question of which modality to begin first. Considerations include: challenges in finding skilled CBT therapists; inconvenience of getting the child to weekly therapy; often-greater expense of CBT from more frequent co-pays; and resistance of the child (and sometimes a parent) to the structure, effort, and discomfort that CBT requires (e.g., in response to planned anxiety exposures). Modality decisions are also influenced by the wish to avoid medication in a child [34], with its under-studied, long-term side effects [35], and concern about relapse after discontinuation [20, 36, 37]; the perception that medication may be less emotionally supportive than psychotherapy; the stigma often associated with medication [38]; or the perception that medication is a “crutch” that instills physical and psychological dependency [39], unlike the skills that CBT aims to impart. Ethnic minorities often have a cultural aversion to medication, particularly for mental health problems, and especially in children [39, 40], but they also tend to view CBT as less feasible for their lifestyle [40], thereby limiting their treatment adherence [41]. Complicating the selection of initial treatment is the absence of data indicating whether sequencing of treatment impacts treatment satisfaction, well-being, peer relationships, parent and family functioning, school functioning, or comorbid psychiatric symptoms [5, 42–44]. Finally, few studies have adequately tested the effectiveness of either treatment modality in the complex and underserved families who are likely disproportionately affected by anxiety disorders. Empirical data for the effectiveness of anxiety treatments in underserved families are particularly sparse for patient-centered outcomes and for the head-to-head comparison of CBT to medication. In the CAMS trial comparing CBT to medication, for example, the patient sample was 78% Caucasian and 75% middle or upper class [10]. A recent AHRQ (Agency for Healthcare Research and Quality) review identified inadequate ethnic and racial diversity as a prominent limitation of prior treatment studies of pediatric anxiety disorders and stressed that characterizing treatment response in underserved ethnic minorities, and identifying modifiers of treatment response, constitute the most pressing needs for future research [20].

Consensus panels formulating treatment guidelines for pediatric anxiety disorders have relied on clinical experience, theory, and findings from observational and controlled trials of CBT or medication conducted in isolation for other purposes. They generally agree that CBT and either SSRI or SNRI pharmacotherapies have the strongest evidence for efficacy [20]. They often recommend CBT as the first-line treatment, but without evidence that it yields better patient outcomes than beginning with medication [44–46]. Many guidelines recommend combining psychological and medication therapy after an unsatisfactory initial treatment response, but without evidence that combined therapy is better than continuing or intensifying the initial treatment. Very few studies have compared combined therapy to either treatment alone [47–49]. In the CAMS study, combined CBT + sertraline improved clinical response more than either modality alone [49], but primarily in those with severe anxiety [50], and not in long-term follow-up [29].

To address these gaps in evidence for development of treatment guidelines for pediatric anxiety disorders, we will conduct a Sequential Multiple Assignment Randomized Trial (SMART) [51, 52], that will develop and test an *Adaptive Intervention –* a set of decision rules for adapting treatment according to a patient’s individual clinical response – for the initial selection of treatment modalities (CBT or SSRI) and their subsequent sequencing, combination, and maintenance in treating pediatric anxiety. These rules will be based on individual patient characteristics -- particularly the patient’s response to initial and subsequent treatment, but also demographic and clinical characteristics -- that optimize treatment response. The SMART design involves randomization of participants at least once, and often more than once, sequentially over the course of the trial. Randomization occurs at critical decision points depending on the patient’s clinical response and is used to provide valid causal inference about the effects of differing interventions that will inform treatment decisions [53, 54]. This design provides a rigorous framework in which to develop evidence-based treatment algorithms. SMART designs make no a priori assumptions about the existence or form of delayed treatment effects [51, 52], making them ideal for developing an adaptive intervention that optimizes outcomes based on patient treatment history [51–54]. The SMART design described here – the first of its kind of which we are aware -- will focus on identifying treatment sequences that optimize patient centered outcomes for pediatric anxiety disorders in ethnically diverse patients from underserved low- and middle-income households.

### Conceptual framework of the SMART design

The conceptual framework for this study is founded on the observation that patients differ in their responses to treatment, presumably due to individual variability in the psychological, biological, cultural, and psycho-physiological factors that shape adaptive and maladaptive anxiety responses to life experiences [55]. Ample evidence documents dysfunction across the neural, cognitive, affective, and behavioral components of anxiety disorders. Less is known about the most effective ways to modify that dysfunction through treatment. Evidence suggests that CBT or medication can modify the cascade of responses to real or perceived threat – CBT by altering behavioral avoidance and information processing related to threat detection and coping [56, 57], and medication by altering neurotransmitter levels that affect the function and structure of brain circuits [58–60]. By experimentally controlling the sequencing of treatment modalities, we aim to advance knowledge about the relative merits of targeting the cognitive and behavioral components of this cascade with CBT, or its biological components more directly with medication.

Individual differences in treatment response likely derive from patient-specific biological, sociological, psychological, historical, and contextual determinants of responses to threat [61]. For a treatment to be most effective, it should be tailored to patient characteristics that influence those determinants. Tailoring should be adapted dynamically, repeatedly over time according to that patient’s individual response to treatment, to produce an adaptive intervention that informs how and when to intervene, and how and when to modify the intervention to optimize long-term outcomes. An adaptive intervention has 4 components: (1) decision stages, each beginning with a decision concerning treatment; each decision stage incorporates (2) treatment options, (3) tailoring variables, and (4) an if/then decision rule. The decision stages, treatment options, tailoring variables, and implementation of decision rules *are all part of the intervention itself* [62]. The overarching aim and function of a SMART design is to construct a high-quality adaptive intervention based on empirical data. A SMART design builds an adaptive intervention that dynamically tailors treatment to individual patient characteristics and evolving therapeutic response to optimize patient outcomes, a process that has been likened conceptually and operationally to the development of a feedback control system for dynamic systems that regulates and optimizes a time-varying study outcome variable [63]. A SMART design offers a considerable practical advantage over more traditional trial designs, in that it addresses simultaneously several research questions and study hypotheses relevant to each decision stage, as well as their interaction representing the sequencing of interventions [64].

#### Patient-centeredness within the SMART design

Adaptive interventions resemble clinical practice, in that different interventions are assigned to different individuals and within individuals over time, with the intervention(s) ideally varying in response to patient needs and patient response [62]. Clinicians, however, often lack the empirical evidence needed to guide such intervention. In pediatric anxiety treatment, limited evidence exists to inform the decision about which treatment to begin with based on a particular patient’s characteristics and context, and what the clinician should do if the chosen treatment is not working. This absence of empirical data for clinical decision-making is a challenge that may be addressed in part via the multistage randomization of a SMART design to inform the development of optimal adaptive interventions, providing an empirical basis for clinical decision-making. The relevance to pediatric anxiety is clear: though substantial evidence supports the effectiveness of both SSRI’s and CBT in treating pediatric anxiety, neither treatment works well for all youth, and much more evidence is needed to guide clinician judgments regarding *which* treatment to use, with *which* patients, and at which points in treatment. The present study will address this gap, providing evidence to inform treatment-personalizing decisions that are required of virtually every clinician treating pediatric anxiety. Findings on the association of baseline individual tailoring and environmental variables with later clinical outcomes can inform the use of individual patient information for initial treatment assignment. Findings of the sequential randomization aspect of the SMART design during treatment can inform clinician decisions about treatment sequencing based on the individual patient’s response to the intervention currently in use, further supporting personalized treatment [53, 54]. Empirical evidence contributing to individualized treatment regimens based on tailoring variables, treatment history, and current response status will have the added advantage of supporting shared decision-making by the child and parents with an empirically-informed clinician [65, 66], ideally improving patient satisfaction, treatment motivation [67], and clinical outcomes [68, 69]. This study will also explore the effects of treatment on long-term patient and family-centered outcomes, such as symptom recurrence, subjective distress and well-being, social relationships, family and school functioning, and potential adverse treatment effects [20, 70]. The findings of this study will therefore inform decisions among patients, families, clinicians, and healthcare leaders about improvements that can be expected in the short- and long-term when treating pediatric anxiety disorders with CBT, medication, or their combination in real-world settings, providing information needed when making critically important treatment choices for individual patients, with a much-needed emphasis on children from underserved and minority populations.

#### Potential for study findings to be adopted into clinical practice and to improve delivery of care

Because adaptive approaches approximate intervention sequences used in clinical practice, they can be used to develop, test, and refine algorithms for clinical decision-making and inform the development and validation of practice guidelines [64]. The adaptive intervention that a SMART design produces can be incorporated into clinical practice more naturally and seamlessly than the findings of a fixed-intervention study [53, 54, 62].

### Hypothesized causal pathways and their theoretical basis in the treatment of pediatric anxiety disorders

**Fig. 1:**
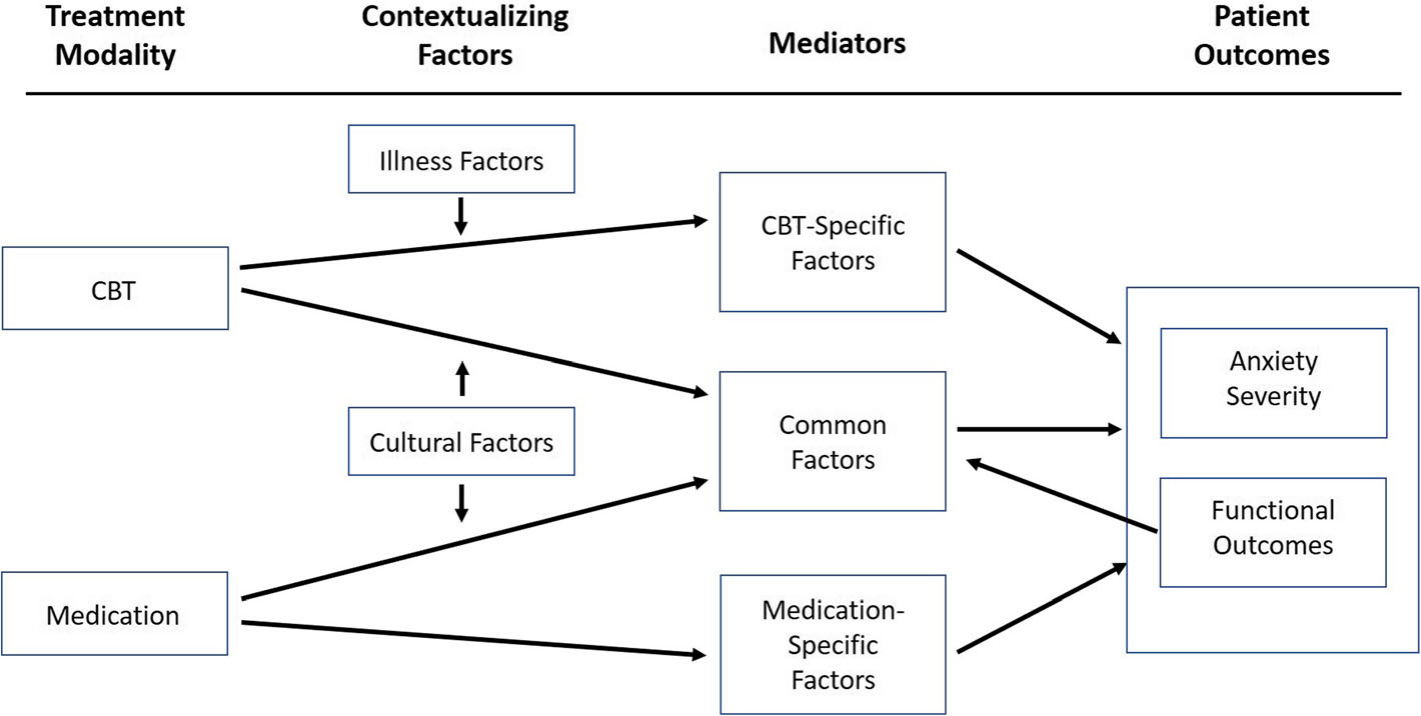
Postulated Causal Pathways CBT-Specific Factors include exposure and development of coping skills. Common factors include the strength of the therapeutic alliance, clinician relatedness, and rewards associated with treatment. Medication-Specific Factors include direct effects of fluoxetine on brain circuits. Functional Outcomes include improved social relationships, better family and academic functioning, and improved self-esteem, which also function as natural reinforcers, or rewards. Vertical arrows from the Contextualizing Factors represent moderation of the association (shown as an oblique arrow) of a treatment modality with the indicated set of mediators. These contextual factors include Illness Factors such as comorbid illnesses, baseline anxiety severity, and family history of anxiety, as well as Cultural Factors, which include ethnicity and SES

Our study population will be predominantly economically disadvantaged, racial and ethnic minority youth, 8–17 years of age. The causal pathways from treatment assignment to clinical outcomes involve factors that are both common and specific to our two interventions (*Coping Cat* CBT and SSRI therapy) **(**Fig. 1**)**. *Coping Cat* CBT, like most psychological or behavioral therapies (henceforth “psychotherapies”), is a complex intervention comprising many elements. Some of those elements are present, to varying degrees, in most or all psychotherapies or even any clinical encounter, including an encounter when prescribing medicine [71, 72]. Some elements are considered unique and specific to CBT [73–75]. We propose to study both the common elements of the clinical encounter as well as elements specific to CBT in this SMART study. Of the many potential factors common to all clinical encounters, two are explicitly components of *Coping Cat* CBT, “alliance-building” and “reward”, and they are at least implicitly components of the psychopharmacological treatments as well **(**Table 1**)**. Two factors are generally considered unique to CBT, “exposure” and “coping efficacy”, or skill building [77, 78]. All four are thought to be important in improving patient outcomes: they are considered to be the “active ingredients” of the complex intervention, and to evolve over the course of the therapy. The extent to which these elements or functions are present in the therapy and increase over time is the extent to which patients are thought to improve. Therefore, we will measure these elements over time in this SMART study and assess their influence on patient outcomes.

**Table 1:** Core Functions and Forms [76] for *Coping Cat*

CORE FUNCTION	CORRESPONDING FORMS
**STAGE 1**	
**Core Function 1: “Build a Collaborative Working Alliance”**	**Psychoeducation:** to develop a shared conceptualization of the tasks and goals of therapy, patients and their families are provided with age-appropriate written materials (as part of the CC manual), and the therapist engages them in discussion about how anxiety can develop and be maintained, and how it can be treated. **Collaborative exercises**: a range of forms are provided to allow therapists to engage in collaborative activity to develop a working alliance. Therapists have flexibility with fidelity to customize the development of the coping FEAR plan (e.g., use of which self-statements) and exposure hierarchy (fear focus, size of steps, timing, use of in vivo vs imaginal exposures). Level of parent and level of youth input into these tasks can be customized to accomplish this core function. **Clinician Relatedness:** through training procedures and supervisory review of videorecordings of patient sessions, supervisors will identify what changes are needed in the clinician’s behavior to optimize empathy and ability to work with the patients collaboratively and in an age-appropriate way; those changes will be modeled by the supervisor, then role-played with the supervisor, who will observe further session recordings to ensure that the appropriate clinician behaviors are established.
**Core Function 2: “Exposure without Avoidance”**	**Exposure Tasks:** multiple sessions of CC are devoted to experiences designed by the therapist in collaboration with the patient, in which the patient is exposed to specific stimuli in the patient’s anxiety hierarchy over time and across successive occasions. Through these experiences the patient learns that the feared stimuli or situations that have been avoided can actually be tolerated, increasingly so, and that feared consequences do not actually occur. The form of these exposures is intended to vary from patient to patient in terms of fear focus, size of steps, timing, use of in vivo vs imaginal exposures and the function may be accomplished by any of these variants.
**Core Function 3: “Develop Coping Efficacy”**	The CC manual provides a wide variety of tools to enhance youths’ and parents’ sense of coping efficacy and the patient’s resulting ability to tolerate and manage negative affect. While all families are provided information and training on all of these tools, the coping FEAR plan may take different forms across patients. It is designed to highlight the tools youths have found most helpful over the course of care. The forms below indicate the range of tools that may be included on the plan in order to accomplish the core function of developing coping efficacy, grouped by domain. **Somatic Management:** tools are provided for identification of somatic cues of anxiety, diaphragmatic breathing, and progressive muscle relaxation. Customization of these exercises is acceptable and expected (e.g., which muscle groups to highlight). Handouts and therapist scripts are available and may be repeated as necessary. **Cognitive Restructuring:** tools are provided to identify thoughts, challenge distorted thinking, analyze automatic thoughts, develop positive self-talk, and enact coping scripts. Handouts and therapist scripts are available and may be repeated as necessary. **Problem Solving:** a problem-solving framework is introduced through therapist scripts, handouts, and in-session activity to provide a rubric to evaluate specific actions for dealing with problems (one homework task to “Show That I Can” (STIC) is assigned each week).
**Core Function 4: “Engage in Reward”**	**Self-monitoring and Reinforcement:** through practice in session, the therapist teaches the child to monitor and evaluate/rate his or her own behavior and to reward self for the effort.
**STAGE 2**	
**Core Function 5: “Intensification”** Continue progression through, and intensification of, exposure	The therapist continues to guide the patient through the patient’s hierarchy to tasks involving the highest levels of difficulty. This is the intensified analogue of Stage 1 Core Functions 2 and 3.
**Core Function 6: “Consolidation”** Consolidate and review previously learned CBT coping skills	Continue practice of learned coping skills. Review accomplishments and rewards.

#### Common factors across both study interventions [71, 72]

These include the quality of the therapeutic alliance, clinician relatedness (especially their degree of empathy, while working with the patients in a collaborative and developmentally appropriate way [79]), and evaluation of response and delivery of rewards associated with improved functioning. *Therapeutic alliance* provides a coherent narrative and conceptual framework through which to understand and address the patient’s suffering within a mutually shared cultural context, thereby providing an explanation for how the patient developed impairing anxiety and what can be done about it. It provides expectancy and hope for change and fosters the fortitude to confront the problems through treatment [80–83]. Prior studies have shown that a better therapeutic alliance precedes and predicts better outcomes, and better outcomes precede and predict an improved therapeutic alliance [71, 73, 84]. Therapeutic alliance will be measured using the *Outcome Rating Scale* [85] **(**Table 2**)**. *Clinician relatedness* includes the personal relationship between therapist and patient, and the extent to which each is genuine with the other. It provides the patient with a connection to a caring and empathic person, which is assumed to be therapeutic in itself, especially for patients who have impoverished social relationships [113–115]. It will be measured using the *Session Rating Scale* [85, 109] **(**Table 2**)**. *Rewards* for improved anxiety outcomes include natural reinforcers, such as improved social relationships, better family and academic functioning, and the improved self-esteem they bring. These rewards in turn strengthen the therapeutic alliance and patient engagement in practices that presumably produced the therapeutic change. They will be measured using patient ratings of life satisfaction [92] and well-being [91], social functioning [92], and school functioning **(**Table 2**)**.

**Table 2:** Patient-Centered Outcomes

DOMAIN	Instrument	Reporter	Ages	# Items	Reliability	Minutes	Timing (week number)
Telehealth Acceptability	Telehealth Acceptability Questionnaire	C,P,Cl	8–17	15	N/A	3	1,12
Telehealth Acceptability	Process Evaluation for Acceptability	C,P,Cl	8–17	4	N/A	10	12
Anxiety Symptoms	SCARED [86]	C,P	7–17	41	.90	9	B,6,12,18,24,Q
Functional Impairment	Child Anxiety Impact Scale [87–89]	C,P	7–17	28	.70–.90	5	B,12,24,Q
Patient-Identified Treatment Needs	Youth Top Problems [90]	C	All	6	69–.88	3	B,12,24,Q
Well-Being	PROMIS Purpose & Meaning Short Form [91]	C,P	All	8	.90–.98	1	B,12,24,Q
Life Satisfaction	NIH Toolbox Life Satisfaction [92]	C,P	3–17	5	.86–.97	3	B,12,24,Q
Self-Efficacy	NIH Toolbox Self-Efficacy [93]	C,P	7–17	10	.90–.92	3	B,12,24,Q
Family Functioning	Family Assessment Device [94]	C,P	12+	12	.69–.84	5	B,12,24,Q
Social Functioning	NIH Toolbox Social Relations & Loneliness [92]	C	7–17	29	.95	10	B,12,24,Q
School Functioning	Grade point average	Report card	N/A	N/A	N/A	N/A	B,12,24,Q
	School attendance	Report card	N/A	N/A	N/A	N/A	B,12,24,Q
	Student Subjective Wellbeing Questionnaire	C	7–17	16	.92	4	B,12,24,Q
Sleep	Sleep Self Report [95]	C	7–12	26	.76	7	B,12,24,Q
	Children’s Sleep Habits Questionnaire [96]	P	4–10	23	.68–.78	7	B,12,24,Q
Emotion Regulation	Brief Version of the Difficulties in Emotion Regulation Scale (DERS-16) [97, 98]	C	11–17	16	.92	2	B,12,24,Q
Coping with Change	Anxiety Control Questionnaire for Children [99, 100]	C	8–18	10	.89	2	B,12,24,Q
Comorbid Psychiatric Symptoms	Child Behavior Checklist [101] Youth Self-Report [102]	C,P	6–17 11–17	83 112	.78–.97 .71–.95	12	B,12,24,Q
	Mood and Feelings Questionnaire [103]	C,P	All	13		5	B,12,24,Q
Side Effects	The Pediatric Side-Effect Checklist	C,P,Cl	All	24	.78	5	B,6,12,18,24,Q
Suicidal Ideation	Columbia-Suicide Severity Rating Scale [104]	C	All	6	.73–.93	2	B,12,24,Q
Family’s Accommodation of Patient’s Symptoms	Family Accommodation Scale-Anxiety [105]	P	All	13	.82–.90	3	B,12,24,Q
Treatment Expectations	Treatment Expectancies [106]	C,P,Cl	All	3	N/A	2	B,M
Service Use	Anxiety Disorders Interview Schedule For DSM-5 (ADIS), the supplemental service form (SSF) [107]	P	All	5	N/A	5	B,12,24,Q
Treatment Preferences	Treatment Acceptability and Preferences [108]	C,P	All	2	.88	5	B,12
Clinician Relatedness & Therapeutic Alliance	Outcome Rating Scale and Session Rating Scale [109]	C,P,Cl	All	8	.87–.96 & .88	1	B,M
Anxious Avoidance	Child Avoidance Measure [110]	C,P	8–18	8	.86–.91	3	B,M,Q
Parent Anxiety & Depression Symptoms	PHQ9 & GAD7 [111, 112]	P	All	16	0.83–0.92 ([111, 112])	3	B,12,24

C=Child-report; P=Parent-report; Cl = Clinician-report

B=Baseline; 6 = Week 6; 12 = Week 12; 18 = Week 18; 24 = Week 24; Q = Quarterly Follow-up; M = Monthly, every 4 weeks during the 24-week trial

**Child Total Time: 93 min**

**Parent Total Time: 78 min**

**Clinician Total time: 10 min**

#### Factors specific to Coping Cat CBT

These include tolerance of exposure to anxiety-provoking stimuli and development of coping efficacy [116] **(**Table 1**)**. *Tolerance of exposure*, with inhibition of an associated avoidance response, is considered the key element in the cognitive behavioral therapy of anxiety disorders [77, 78]. Exposure is thought to combat fear and avoidance via one or both of two learning processes, habituation and extinction learning (also termed inhibitory learning). *Habituation* is a form of non-associative learning in which repeated exposure to the feared stimulus or situation produces a transitory weakening of fear responses, with a higher frequency of exposure producing greater habituation, and a greater duration between exposures producing a greater return to pre-exposure levels of fear response [117]. Habituation also produces a steady decline in neural response to the feared stimulus. *Extinction learning* refers to the reduction or extinction of the fear response following repeated exposure to specific fear-eliciting situations in the absence of the aversive consequences with which it was previously paired. This exposure generates new learning of safety-based associations that inhibit former fear response associations to the feared stimulus [77, 78, 118], thereby either extinguishing the fear response or enabling a level of fear tolerance that reduces anxious distress. Extinction activates neural pathways that inhibit or modulate emotional responses and avoidance of the feared stimulus [57].

Exposure also promotes the development of cognitive, emotion regulation, and behavioral skills for coping more effectively with fear, termed *coping efficacy.* Coping efficacy diminishes the conditioned fear response and provides the opportunity for new extinction learning, with an attendant inhibition of maladaptive avoidance. Practicing these skills in different fear-inducing contexts supports generalization of inhibitory learning and stronger extinction of fear responses [78]. Of note, both time spent in exposure tasks, and level of difficulty of the exposures tolerated, predicted better outcomes to *Coping Cat* within the CAMS sample [119]. These findings directly informed our decision to have additional exposure practice and mastery be the focus of our Phase 2 CBT optimization protocol. In addition, efficacy measures in the CAMS trial outperformed specific measures of cognitive change in mediational models on Coping Cat effects, suggesting that development of coping efficacy may mediate CBT outcomes [120].

We will obtain after each CBT session a therapist-rated measure of 3 key facets of exposure practice (quantity, difficulty level, and mastery of exposure tasks) [119], which we will use to craft an index of tolerance to exposure – a CBT-specific factor in effecting patient outcomes. As a complement to this in-session measure of tolerance of exposure, we will also obtain at baseline and then every fourth session the parent version of the 8-item *Child Anxiety Avoidance Scales* [110], a measure of the evolving real-world CBT skill set outside of session that assesses the reduction of anxious avoidance when presented with threatening stimuli. Finally, we will use our measure of Self-efficacy from the NIH toolbox measure of Life-Satistfaction as a proxy for coping efficacy to test this CBT-specfic pathway. We expect that measures of each of the common factors will independently and positively associate with outcomes in our study for both *Coping Cat* and medication therapies [72, 82, 121], and we expect that the greater therapeutic alliance anticipated with CBT compared with medication therapy will partially mediate differences in clinical outcomes across the two treatment modalities. We also expect that measures of the CBT *specific factors* -- exposure tolerance and coping efficacy -- will positively associate with clinical outcomes in response to *Coping Cat* CBT.

#### Factors specific to fluoxetine administration

We do not yet know precisely how medication, including fluoxetine and other SSRIs, improve symptoms in pediatric anxiety disorder. It is highly probable, however, that those mechanisms are initiated by the effects that medication has on altering neurotransmitter levels, which in turn affect the function and structure of brain circuits [58–60]. SSRIs inhibit the presynaptic reuptake of serotonin after its release into the synaptic cleft, which in turn, over time, desensitizes HT1a serotonin receptors on or near the cell body of the presynaptic neuron [122]. Desensitization of these presynaptic serotonin receptors then increases impulse flow in the presynaptic neuron and ultimately increases serotonin concentrations in the synaptic cleft. Serotonin HT1b receptors on the presynaptic terminal then also desensitize, further increasing presynaptic transmission and increasing the serotonin available to stimulate postsynaptic neurons. Precisely how an increase in postsynaptic serotonergic signaling improves anxiety symptoms is unknown, but presumably therapeutic effects pertain to increased serotonergic tone in the multiple and widely distributed neural systems that serotonin influences, including arousal pathways from the midbrain raphe to prefrontal cortex, or from the midbrain to basal ganglia, mesolimbic cortex and hippocampus, or hypothalamus [122]. Most brain imaging studies of the effects of SSRI or SNRIs on brain structure and function have been poorly controlled and naturalistic. The few imaging studies that have been combined with an RCT design to provide stronger causal inference of the effects of these medications have shown that they normalize pre-existing abnormalities in brain structure [59], function [60], and metabolism [123]. We will not be able to measure these features in all study participants, though we plan to offer MRI scanning immediately before starting treatment, as well as at weeks 12 and 24, to any willing participants within a PCORI- and IRB-approved add-on study to our SMART design. We anticipate that fluoxetine will normalize pre-existing differences from healthy controls in measures of brain structure, function, and metabolism in youth with anxiety disorders, as found in the prior RCT studies for medication effects [59, 60, 123], and that CBT-induced changes in brain structure, function, and metabolism in brain regions that subserve extinction and inhibitory learning (dorsal frontal, basal ganglia, and ventromedial prefrontal cortex) will uniquely associate positively with improvements in patient outcomes [57].

#### Contextualizing factors

Also termed patient “tailoring variables”, these factors modify treatment response. Candidates for tailoring variables include baseline individual, family, and context characteristics, some of which relate directly to child anxiety and its clinical portrait: ethnicity [30], SES [32], past treatment response [29, 31], and family history of anxiety; and variables that are potentially modifiable, such as overall symptom severity [29, 31], functional impairment [29, 31], the nature and severity of comorbid illnesses, treatment fidelity and adherence, and treatment setting (community or university; primary pediatric or specialty mental health clinic).

We theorize that demographic characteristics such as ethnicity [30] and SES [32] will function as *cultural factors* that will influence the nonspecific factors of therapeutic alliance, clinician relatedness, and treatment adherence. Data from the CAMS trial support these hypotheses. In CAMS, African-American youths attended fewer CBT and medication management sessions, and they were rated by therapists as less involved and compliant with treatment and, possibly as a consequence, as showing a lower level of mastery of CBT concepts [41]. Controlling for these process factors and SES eliminated racial differences in outcome. Similarly, patient nonadherence (poor attendance, low homework completion, poor compliance in session) was associated with number of parents present in the home (with the best outcomes for two-parent families), although indices of nonadherence varied in their power to predict clinical outcomes [124]. In contrast, we expect the modifying effects of *illness-related factors*, such as depression and other comorbid illnesses, baseline anxiety severity, and family history of anxiety, will operate through their impact on the *Coping Cat*-specific factors, exposure and development of coping skills [29, 31, 119]. Table 2 specifies how we will measure each of these contextualizing constructs.

## Methods

### Specific aims

**Fig. 2:**
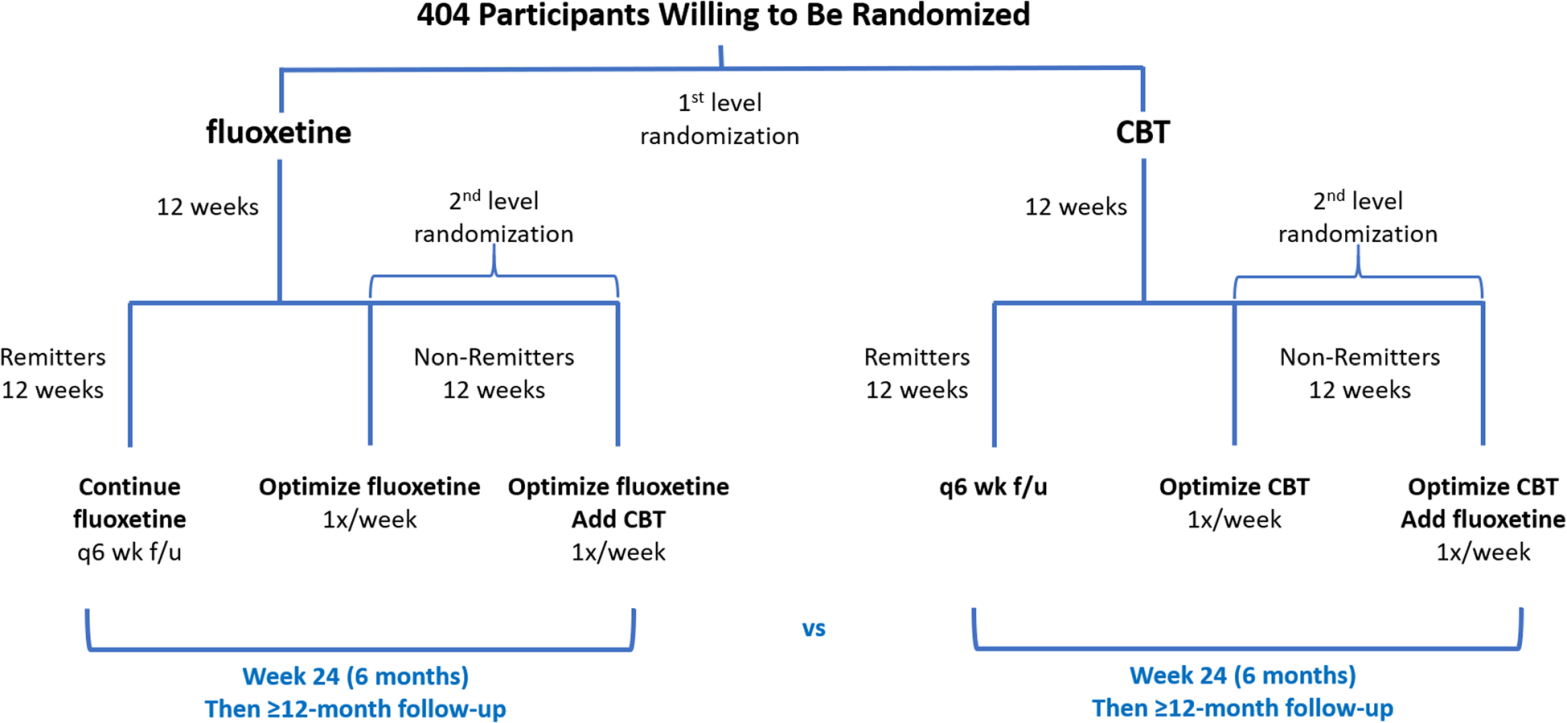
Schematic of Study Design

1. In 404 predominantly underserved, ethnic minority children who have a DSM-5 anxiety disorder, we will conduct a 24-week Sequential Multiple Assignment Randomized Trial (SMART) **(**Fig. 2**)** with the following aims:
  (a) We will assess whether beginning with Cognitive Behavioral Therapy (CBT) or fluoxetine medication is more effective in improving youth-rated anxiety symptoms over the 24-week intervention (“Main Effect 1”)
  (b) If the initial intervention fails to induce clinical remission by week 12, we will assess whether optimizing the initial treatment modality alone, or adding the other modality while optimizing the first, yields better symptom improvement by week 24 (“Main Effect 2”)
  (c) We will assess whether one sequence of treatment modalities – i.e., CBT➔CBT; CBT➔CBT + med; med➔med; med➔med + CBT -- is significantly better or worse than predicted from the two main effects.
  (d) We will assess the stability of treatment response for ≥12 months following completion of the 24-week trial.
2. We will explore the moderating effects of patient characteristics on Main Effects 1 and 2, and on their interaction, to identify tailoring variables that will support personalized interventions for selection of initial and subsequent sequencing of treatment modalities for pediatric anxiety disorders.
3. We will explore the differential treatment effects of our interventions on other patient centered outcomes, including: well-being; life-satisfaction; self-efficacy; family, social, and school functioning; sleep; emotion-regulation; coping with change; comorbid psychiatric symptoms; and adverse treatment effects.

#### Hypotheses for Aim 1

(1a) Anxiety symptoms will improve more in children who begin treatment with fluoxetine than in those who begin with CBT. We base this hypothesis on several considerations. First, medication in the CAMS study exhibited a small, non-significant advantage over CBT on ratings from the Pediatric Anxiety Rating Scale [49]. Ethnic minorities also demonstrated poorer adherence to CBT in the CAMS trial [41, 124], and these youth will be strongly represented in our study population. In addition, unlike CAMS and most prior treatment studies for pediatric anxiety, we are not excluding comorbid depression, which can exacerbate anxiety [125]. Because fluoxetine is helpful in treating both pediatric anxiety and depression, we anticipate added benefits to fluoxetine in treating anxiety symptoms via its effects on comorbid depression; (1b) Based on the greater response to combination therapy than to monotherapies in the CAMS study [49], we hypothesize that in children for whom initial treatment fails to produce remission by week 12, anxiety symptoms will improve more when the alternative treatment intervention is added to optimization of the initial intervention, compared to optimizing the initial intervention alone.

### Study design

This will be a single-blind [126, 127] SMART design [51, 52] of 24-week treatment duration that will employ two sequential levels of randomization, one in each of two 12-week stages of the study **(**Fig. 2**)**.

#### Stage 1

404 children ages 8–17 who have an anxiety disorder will be randomized 1:1 to receive 12 weeks of either the medication fluoxetine in upward-titrated dosages or weekly CBT implemented using the *Coping Cat* CBT model [128].

#### Stage 2

All participants who do not remit in Stage 1 will be randomized 1:1 to either (1) optimization of their Stage 1 treatment, or (2) optimization of their Stage 1 treatment and addition of the other treatment modality (Fig. 2). Study assessments will be obtained at baseline, week 12, and week 24. A small subset of measures (SCARED-41, and the Pediatric Side Effect Questionnaire) will also be obtained at weeks 6 and 18. Participants who remit during the first 12 weeks of treatment will continue maintenance-level therapy with the single-modality treatment received in Stage 1. If a participant who achieved clinical remission during the first 12 weeks of treatment relapses at the week 18 study assessment (defined as having a SCARED total score of ≥25), the participant will be referred to the treating clinician to restart or slightly intensify the previous treatment assigned in Stage 1 of the study. Following conclusion of the 24-week trial, we will follow and obtain all the same study assessments in all participants quarterly for at least 12 months [44, 126, 127].

Our criteria for remission during the trial will include (a) a youth SCARED-41 score less than diagnostic threshold for any single anxiety disorder, together with (b) a total youth SCARED-41 score < 10, *and* (c) a score of ≤8 on the CAIS (Child Anxiety Impact Scale). The CAIS score predicted remission in the CAMS trial [87, 88], and the CAIS was sensitive to change over an 8-week RCT [89]. These remission criteria are intended to represent few residual anxiety symptoms together with minimal functional impairment. The stringency of these remission criteria is based on the reviewed prior studies indicating the importance of achieving diagnostic remission, with few residual symptoms and minimal functional impairment, in optimizing long-term clinical outcomes. All participants who do not meet these remission criteria will be randomized (1:1) for Stage 2 treatment, either to (1) optimization of their Stage 1 treatment, or (2) optimization of their Stage 1 treatment and addition of the other treatment modality. Our use of patient-report measures to define remission at Week 12 will allow us to quickly evaluate clinical status and move to either maintenance treatment or second-stage randomization with minimum disruption in care.

The Stage 2 interventions mirror current best clinical practice in several ways. First, no viable treatment option beyond CBT, SSRI, or their combination exists for partial or non-responders. Second, *Coping Cat* often requires more than the manualized weeks of treatment, particularly in lower income and minority populations, who may face additional challenges with regard to missed appointments and completion of homework assignments [40, 41]. *Coping Cat* also has 2 distinct phases of implementation, with phase 1 comprising psychoeducation, coping skills development, and initial introduction to exposure, and phase 2 intensifying treatment through more challenging and repeated exposure activities, which map well onto our 2 SMART stages. Third, optimization of medication dosing often is not complete within the first 12 weeks, particularly for younger participants, and the effects of any given dose may not fully manifest for 12 weeks or more [21, 22, 129]; thus, the effects of Stage 1 dose increases may not manifest until Stage 2. As SMART designs make no a priori assumptions about the existence or form of these delayed treatment effects [51, 52], they are ideal for developing an adaptive intervention that optimizes patient outcomes based on treatment in Stage 1 [51–54].

#### Comparators

**Fig. 3:**
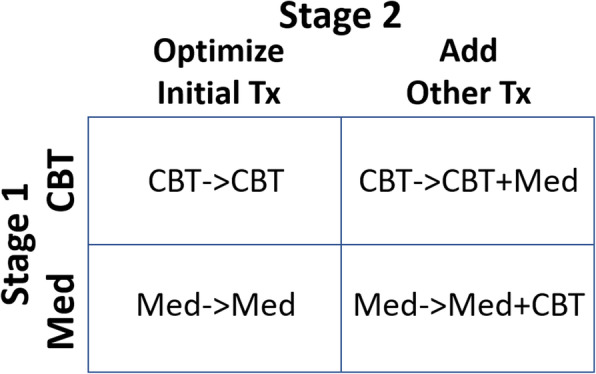
Factorial Structure

Our 2-stage randomization crosses two Stage 1 interventions with two Stage 2 interventions, creating a factorial structure **(**Fig. 3**)**.

*Testing Effect 1* compares the effectiveness of CBT with fluoxetine medication in improving patient-centered outcomes over the 24-week trial. It answers the question that all patients, parents, and clinicians ask when beginning treatment for an anxiety disorder: “Is it better to begin with CBT or medication?”

*Testing Effect 2* compares 2 interventions in their effects on patient centered outcomes by week 24 in patients for whom the Stage 1 intervention fails to induce clinical remission by week 12: (a) optimizing the initial treatment modality alone; and (b) adding the other modality to optimization of the first. It answers the question that arises soon thereafter in the treatment of most patients: “If clinical response to the initial treatment is less than ideal, is it better to continue and optimize that initial treatment, or to add the other treatment modality to the first?”

*The interaction* effect assesses whether one of the 4 treatment sequences – CBT➔optimized CBT; CBT➔optimized CBT + medication; medication➔optimized medication; medication➔ optimized medication+CBT – is significantly better or worse than predicted from the two main effects alone. It should be noted that these interaction effects are not the same interactions that might be addressed in a classical factorial trial design because the treatments and groups are not concurrent, nor are the same conditions present for combining groups [130]. Nevertheless, testing this effect will address another question that arises during clinical treatment: “Which sequence of treatments will produce the best response – fully optimized CBT alone; CBT with the addition of medication; fully optimized medication alone; or optimized medication with the addition of CBT?” It also answers the question of whether the effects of combined treatment are additive or multiplicative relative to the effects of monotherapy.

#### Study population and setting

Los Angeles County (LAC) is one of the most racially, ethnically, and socioeconomically diverse regions in the world [131]. Our study population will be recruited from our vast LAC clinical network serving predominantly underserved, impoverished, and ethnic minorities. Locating these recruitment and treatment sites in LAC provides great efficiency in study infrastructure while simultaneously yielding a remarkable diversity of settings including primary pediatrics care (CHLA Care Network, AltaMed sites), a private health care plan (Kaiser site), a free-standing children’s hospital (CHLA site), and community mental health clinics serving primarily youth with Medi-Cal insurance, the federal Medicaid program in California (Hathaway-Sycamore, UCEDD, LAC+USC, Children’s Bureau of Southern California sites) or commercial insurance (LifeStance Health California site). The total number of patients seen in our age range across these 9 sites is > 300,761 annually (601,522 over 2 years of recruitment), and 2.9% of them already carry a diagnosis of anxiety disorder in their electronic medical record. We would need to recruit only 0.49% of all patients already diagnosed with an anxiety disorder across these sites to meet our target total of 404. We expect several times more than this number of patients to be identified through systematic screening efforts at each site, further enhancing the feasibility of recruiting our target number.

*All clinical care components of the study, including CBT and medication management, will be paid through the standard mechanisms at each clinic.* At all participating sites, with the exception of Kaiser, LifeStance Health, and CHLA’s care network facilities, Medi-Cal pays for services without patient co-pays. Even these exceptional facilities have ~ 20% of their patients with Medi-Cal. Those with private insurance have small co-pays that families can usually afford.

### Study participants

We will aim for a sample that represents best practice in high quality clinical care by including most comorbidities. We will not exclude based on past diagnoses or treatment, in part because current diagnosis and treatment will be the focus of this study, and in part because it will not be possible to ascertain the validity of past diagnoses or quality of past treatments reported by patients or parents. Not excluding on these past variables will also enhance the generalizability of this study’s findings.

#### Inclusion criteria

- Patient at one of our clinical study sites
- Age 8–17 inclusive (this age range allows the same CBT intervention and outcome measures for all participants),
- SCARED-5 screening score ≥ 3 (see “Screening” below) and score ≥ 25 on the SCARED-41,
- A diagnosed anxiety disorder (generalized anxiety, separation anxiety, social anxiety, panic disorder) on the clinician-administered Schedule for Affective Disorders and Schizophrenia for School-Aged Children, Computerized version (K-SADS-COMP)
- CAIS (Child Anxiety Impact Scale) > 8 (representing at least moderately severe illness).
- We will include patients and at least one parent/caregiver who are fluent in either English or Spanish to ensure accurate assessment, as most standardized measures are normed only in those languages, and sites have limited capacity to provide translation services in other languages. Trial staff and clinicians will be fluent in both languages, and all selected outcome measures will have been previously validated in both languages. Based on comprehensive survey data, we estimate that only 0.3% of families at our sites will not have one parent/caregiver who speaks either English or Spanish.

#### Exclusion criteria

- Patients currently receiving psychotherapy. Prior psychotherapy of any kind, including CBT, is not exclusionary.
- Patients currently receiving an SSRI, SNRI, or benzodiazepine. Prior use of any of these medications is not exclusionary.
- Patients with a severe neurological disorder or unstable medical condition, as determined by medical chart and medical history review by the site director and Principal Investigator
- Females who are pregnant or sexually active but not using an effective method of birth control (potential adverse fetal effects of medication [132])
- Patients who have taken Monoamine Oxidase Inhibitors (MAOIs), pimozide, thioridazine, olanzapine, tricyclic antidepressants (TCAs), antipsychotics such as haloperidol and clozapine, anticonvulsants such as phenytoin and carbamazepine within 2 weeks prior to starting the study, as these can interfere with metabolism of fluoxetine
- Columbia-Suicide Severity Rating Scale [104, 133] score of:
  - 3 AND “access to crisis level support is unavailable”, OR
  - 4 if “frequency, duration, and deterrent” all = 1 AND treatment in a specialty mental health clinic is not available, OR
  - 4 if “frequency, duration, or deterrents” are > 1, OR
  - 5
- To map onto the cognitive and socio-emotional demands of the CBT protocol, we will exclude youths who are likely to be functioning at a developmental level outside the minimum age for the treatment manual (age 8): namely, youths who are placed outside of a general education classroom for > 50% of the school day or require a one-on-one classroom aide to maintain placement in a general education class, or are performing academically below the 3rd grade level in reading and language arts.
- Current, clinically significant Obsessive-Compulsive Disorder (OCD) or Post-Traumatic Stress Disorder (PTSD). Coping Cat CBT was not designed for, nor is it an appropriate treatment for, OCD or PTSD. The most appropriate evidence-based treatments for these two disorders are Exposure and Response Prevention for OCD, and Trauma-Focused CBT for PTSD. Those two treatments differ markedly from the Coping Cat procedures clinicians will be taught in our study. It would be clinically inappropriate and arguably unethical to train clinicians in Coping Cat and then assign study patients to them who have disorders that cannot be effectively treated with Coping Cat. Moreover, clinically significant PTSD usually requires combined medication and Trauma-Focused CBT from the outset, making these youth inappropriate for this clinical trial in which participants are initially assigned to a monotherapy.
  - OCD will be diagnosed using the clinician-administered KSADS-COMP.
  - PTSD will be assessed using the Lang Child Trauma Screen [134, 135]. To optimize the representativeness of our sample, we desire a very low false positive rate for a screener – i.e., we want a screener cutoff with high specificity for diagnosing PTSD. ROC curves for this screener [134, 135] indicate that by requiring a score of ≥10 on the parent report for child reaction to past trauma, we will miss diagnosing approximately 22% of true PTSD cases but we will not exclude any who are incorrectly diagnosed. Therefore, we will exclude youth for whom (a) either the parent OR the child reports a history of trauma on this screener, AND (b) the parent reaction score is ≥10
- Youth currently in foster care. We have extensively explored with the CHLA IRB and our clinical sites the procedures required to enroll these youth in research treatment studies. This includes permission of the court (both the patient’s court-appointed attorney and the judge who oversees the case), the Department of Children & Family Services, and biological parents (depending on the status of the case and who has medical rights), a process that requires institutional lawyers and that can take many months, requiring an extraordinary amount of time and effort on the part of the study team. Potential treatment issues that include the need to obtain additional court approvals when randomized to the medication arm will also likely create a delay in care and systematically affect the medication arms of our trial. Finally, participation requires the de facto permission of the foster parents to ensure the youth will attend clinical appointments. Although we would like to include youth in foster care, it simply is not feasible.

#### Non-randomized, treatment-as-usual (TAU) group

It is possible that medication and CBT will produce similar therapeutic responses in our patient population. We would like to be able to estimate the effect of each treatment and each treatment sequence in our SMART design against usual treatment in real-world settings. Therefore, we will include a non-randomized TAU group consisting of patients who satisfy all study eligibility criteria, pass the 3-level screen, an decline participation in the randomized trial, but who consent to participate in a truncated study measurement sequence. In addition to the eligibility measures for these patients, we will acquire a small subset of measures (SCARED-41, CAIS, CBCL, treatment expectations, ADIS Supplemental Service Form) at the same time points as in the randomized group – at baseline and weeks 6, 12, 18, and 24, as well as the quarterly follow-up assessments thereafter.

#### Screening

We anticipate screening 80% of newly registered and current patients (ages 8–17) for anxiety disorders at our 9 study sites. We will employ a 3-stage screening process. In Stage 1 Screening, we will administer the 5-item version of the parent and child SCARED [86, 136, 137] to patients presenting to any of our outpatient services at all our performance sites. A cut-off score ≥ 3 discriminates “anxiety” from “non-anxiety” patients with 74% sensitivity and 73% specificity [86] and has demonstrated utility to screen youth at risk for anxiety across primary care settings [138]. We will regard a score of ≥3 on either the parent or child SCARED-5 as a positive screen. The screener will request permission from the parent to allow the clinic to share the parent contact information with our study team if the child screens positive for anxiety. In Stage 2 Screening, youth who screen positive on either the parent or child 5-item SCARED will complete the 41-item SCARED. Those who score ≥ 25 [139, 140] on the total score for the parent or child SCARED will undergo further characterization for study eligibility and possible recruitment. In Stage 3 Screening, youth and their parents who pass Stage 2 screening will be administered several additional assessments to complete determination of study eligibility. These will include the clinician-administered KSADS-COMP (for diagnoses of anxiety disorders and OCD), Child Trauma Screen (for PTSD), Columbia-Suicide Severity Rating Scale, CAIS (for functional impairment), and a patient profile (for medical illnesses, treatment, and demographic information).

#### Recruitment

For patients satisfying all eligibility criteria, a study coordinator will contact the parent and patient, describe the study (including randomization requirements), address questions, and, if the parent and patient agree to participate, schedule a baseline assessment and initial clinical visit. Formal written consent and assent will be obtained in person in a private area or online, over the telephone, or by mail at the baseline assessment. When consenting in person is not an option, we will consent over the phone or video conference.

We will attend carefully to elements of study design and execution that enhance recruitment. Participants are often concerned about being randomized to a less effective treatment [141, 142], but here the 2 treatment arms will be in true clinical equipoise [143]. Patients will not face the possibility of being randomized to an ineffective or minimally effective treatment. This was important to the parents in our community engagement activities, who enthusiastically supported our proposed SMART design. Outcomes will be patient-centered [141], in many cases the study will be presented by their primary clinician [141, 144, 145], treatment expectations and preferences will be explored [146], informed consent will be presented simply, confidently [147], and in lay language [141, 142, 144, 145], most clinic/study visits will be scheduled after school or on weekends [144, 148], study demands will be kept to a minimum [141, 142], and patients and parents will not be blind to treatment assignment [141, 142, 145]. A large majority of participants will have Medi-Cal insurance, which does not require co-payments, so participation should not entail a financial burden [149]. We will employ the principles of QuinteT Recruitment Intervention methods, which comprise in-depth, real-time investigation of recruitment obstacles, participation, attendance, and motivation, followed by implementation of tailored strategies to address identified challenges across all treatment arms as the trial proceeds [143, 150]. We will record demographics and screening measures of all screened patients to compare those who do and do not choose to participate, to assess the representativeness of our study sample. We estimate an attrition of 20% over the 24-week RCT, yielding 323 treatment completers. If attrition proves higher, we will increase numbers recruited to ensure we achieve at least that number of completers. Our patient numbers, clinician staffing, and research coordinator burden will readily support this increased recruitment.

#### Retention

These efforts will begin with informed consent. Developing a trusting personal relationship with the family is vitally important for study adherence and ongoing participation, and the treatment provided during the study will help to build those relationships. We will strive to assign a specific research assistant to each patient for the duration of participation. If study appointments are missed, the family will be contacted and the visit promptly rescheduled. If unable to reach them during regular business hours, we will attempt contact at varying days and hours. Cards and small presents will be provided on holidays and birthdays. We will collect comprehensive tracking and location information, including home and work addresses, email addresses, social media contacts, Medicaid and social security numbers of parents and participants, and phone numbers of relatives and others with close relationships with the family. We will conduct quick monthly check-ins with all participants (e.g., text message, social media direct message) to foster our relationship and ensure contact information is current; participants will receive $5 for each check-in completed. When not found at their last-known residence, staff will review change-of-address reports and contact next-of-kin or participant-identified friends. We will keep a record of clinics attended by our participants and, as necessary and with permission, use the clinics to maintain contact. We will compensate participants appropriately in the randomized trial for each research assessment, escalating compensation with time after the lengthier baseline visit to encourage continuing participation ($25 for baseline, $5 for week 6, $20 for week 12, $5 for week 18, $65 for week 24 assessments, $5.00 for each monthly check-in, and $20 for each of the quarterly follow-ups after the 24-week trial). Participants in the non-randomized (TAU) cohort will received $5 for their assessments at each time point.

### Community and stakeholder engagement

One of our core values is community engagement. We are committed to ensuring that patients, caregivers, clinicians, and other healthcare stakeholders play a meaningful role throughout the entire research process. We see our patients and other healthcare stakeholders as equitable partners—not simply as research subjects—and we plan to leverage their lived experiences and expertise to ensure this research is patient centered, relevant, and ultimately useful. Their values, priorities, and preferences are of paramount importance, and so they have been full partners in developing this study design and in selecting its measures.

#### Our community partners

This research will be conducted in Los Angeles County, which is more populous than 42 states and more racially/ethnically, linguistically, culturally, religiously, and socioeconomically diverse than any other city or county in the world. We have deep expertise conducting culturally tailored community engagement that fully embraces this diversity, and in conducting research in partnership with underserved, under-represented, and high disparity populations. We define *patient partners* as including teenagers (≥ age 12 years); parents, guardians, caregivers; parent advocacy/support groups; and local, state, and national organizations and advocacy groups (e.g., National Alliance on Mental Health, with a California chapter and local chapters throughout Los Angeles). We define *stakeholder partners* as including pediatricians and community primary care physicians, hospitals, health/mental health systems, teachers and educators; purchasers, and policy makers (including local health-related foundations).

#### Leveraging our Southern California Clinical and Translational Science Institute (SC CTSI)

We will leverage the resources, services, and infrastructure available through our SC CTSI and its Community Engagement (CE) Program. The CTSI was created to engage vulnerable communities in clinical and translational research; facilitate academic-community partnerships to ensure patient and community engagement in research; and develop, evaluate, and disseminate novel approaches to engaging diverse populations in research. The SC CTSI will offer a range of resources to this project, including access to community health workers who have deep roots and expertise working in Latino (*promotoras)*, African American (*Cultural Brokers)*, and Asian American communities.

#### Community engagement to inform study design and methods

We conducted extensive engagement activities with patients, parents, and caregivers, healthcare providers, and other stakeholder groups, to ensure our research incorporates their voice and reflects their interests in a meaningful way. These activities were conducted in both English and Spanish, in safe spaces (churches, schools, libraries, community health centers) located in East, South, and Central Los Angeles. A brief description of these engagement activities, and important feedback obtained during each, follows:

A. Community Listening Sessions. We hosted a well-attended Community Listening Session focused on pediatric anxiety, during which parents shared their experiences and discussed the challenges associated with raising a child or adolescent with an anxiety disorder. Parents told us how they would want to be involved in this study and offered considerable input as to how we might collectively disseminate research findings to ensure uptake by patients and other stakeholders.
B. Our Community/Our Health Los Angeles (OC/OH-LA). OC/OH-LA is a CTSI community engagement strategy to foster ongoing conversation between health researchers and community members. We hosted an OC/OH-LA session, which was attended by 45 parents, grandparents, and mental health community workers from South L.A. We provided an overview of pediatric anxiety and approaches to evidence-based treatment, followed by questions and discussion. Parents discussed the shame that youth with a mental health condition feel, the secrecy they keep, and how this shame/stigma creates a significant barrier to accessing treatment services. Parents also expressed their fears, misperceptions, and general lack of awareness of the safety, efficacy, and long-term effects of medications to treat anxiety. This feedback deeply informed our planned recruitment and retention efforts. Moreover, it suggests that our dissemination efforts targeted to consumers of care will need to address, head-on, both stigma and fear of medication if our goal to encourage uptake of the study findings is to be achieved.
C. Focus Groups with Parents and Teenagers to Identify Patient-Centered Outcomes. We conducted focus groups with parents/caregivers and, separately, teenagers living with anxiety, referred from mental health service organizations. Parents and their teenage children identified outcomes that concerned them most, as listed in “*Patient Centered Outcomes*” **(**Table 2**)**. They also echoed the OC/OH-LA session, calling for outreach efforts to address stigma and medication concerns. This feedback drove decisions about study outcomes and the scales used to assess them.
D. Research 101. Another SC CTSI community engagement activity is a *Research 101* training manual and curriculum entitled, “Engaging Communities in Research, the Fundamentals of Research,” which presents the fundamentals of research, the importance of increasing diversity in research, and potential myths and barriers to participation. We offered Research 101 to a group of parents and caretakers. In general, we heard that parents have many fears and misperceptions about research and the intentions of researchers. Parents also expressed concern about randomization as potentially “unfair” if treatment is withheld. Following Research 101, parents demonstrated increased knowledge about research, study methods, and designs. Parents also appreciated and understood how, in our proposed study, treatment will not be withheld but rather randomization will occur to evaluate the effectiveness of two gold-standard treatment modalities; they were highly supportive of this design, suggesting that we offer Research 101 to our Parent Advisory Council members (described below) and an abbreviated version of Research 101 to parents of our participants before beginning the study, to help them understand and feel more comfortable with their child’s participation. We believe this will also improve participant retention. We will include information about research methods in our dissemination materials (e.g., newsletters, brief summary reports) throughout the study and at its conclusion to encourage uptake of study findings.
E. Engaging Mental Health Providers & Mental Health Support and Advocacy Groups. We met with mental health support and advocacy organizations to solicit suggestions about study design and our approach to engagement. Participants included the Director of the County’s Department of Mental Health and the President of NAMI California. Other organizations that we have begun to engage and will continue to work with include: the American Academy of Child and Adolescent Psychiatry; National Federation of Families for Children’s Mental Health; Mental Health America; Anxiety and Depression Association of America; United Advocates for Children and Families; California Mental Health Advocates for Children and Youth; Los Angeles Unified School District; Pacific Clinics; Young Minds Advocacy; and our study’s partner sites. We will also work with 2 youth-focused organizations: (1) International Children’s Advisory Network (iCAN), a worldwide consortium of children’s advisory groups known as Kids Impacting Disease through Science (KIDS); and (2) Young Person’s Advisory Groups (YPAGS), youth groups working to provide a voice for children and families in research. These organizations will help us develop our own KIDS and YPAGS for this project. Also, we will partner with WeRiseLA; this organization seeks to use art and community-building to transform the mental health care system and to foster mental health and wellbeing as a civil right.

#### Community engagement activities

Based on the feedback obtained from parents, parent advocates, and mental health providers, we will employ the following structure and activities before, during, and after our study:

#### Advisory committees

We will establish 2 advisory groups: 1) a Parent Leadership Council (PLC), and 2) a Youth Leadership Council (YLC). Both will provide ongoing feedback and assistance to: refine study questions and research methods; assist with outreach; develop recruitment and retention protocols; refine assessment procedures and measures; troubleshoot challenges; review performance; and interpret and disseminate findings. Each group will meet bi-monthly and will be facilitated by the Community Engagement team. Both will report directly to the study PI; the PI and research team will attend these meetings to ensure members’ ongoing, meaningful input. Employing community-based participatory research principles relevant to partnership development, we will work continually to foster bi-directional collaborations and partnerships. We will ensure that our partners are included in all stages of the study, we will integrate knowledge and action that is beneficial to all partners, and we will promote a co-learning and an empowering process.^15^ We chose to create 2 separate committees based on feedback received from parents and youth; each wanted a “safe space” in which to share their thoughts and opinions, without possible “judgment” from the other. We will provide each of these groups regular updates on study progress, report challenges, brainstorm solutions, share successes, discuss/interpret findings, and assist with dissemination. A standing meeting agenda will include capacity building (e.g. how to interpret a conceptual model, how to read a table of data) to create an environment of reciprocal relationships and co-learning; discussions about the best way to implement the study – how to engage, recruit, and retain patients/parents; how to message the study in a culturally relevant/tailored way to the community; culturally appropriate staffing for the project; ideas for co-learning opportunities; and ongoing review of recruitment techniques and retention methods. The Southern California Clinical and Translational Science Institute will offer an educational workshop, called *Research 101*, to our two stakeholder advisory committees. *Research 101* was developed for lay audiences to a) address myths and fears about research, b) increase scientific literacy about clinical research is conducted, and c) offer information about study participants’ rights. Advisory committees will meet remotely throughout the course of the study using a HIPAA-compliant communications platform (e.g., Zoom). The following provides a brief description of each advisory committee.

Parent Leadership Council (PLC) The PLC will be established so parents and caregivers can provide input to the research team. We will work with United Advocates for Children and Families Parent Leadership Institute to provide training and support to parent members, to help them further develop leadership and advocacy skills. An ongoing agenda item will be to review content and format for a quarterly project newsletter for participants and their families. Consistent with emerging best practices for conducting focus groups via teleconference during the pandemic [151, 152], the PLC will include 8–10 parent representatives from local provider networks, parent support groups, parents from our performance sites, and other organizations. This Council will meet bimonthly.

Youth Leadership Council (YLC) The YLC will be established and included as equal partners in study design and implementation, based on feedback from CHLA’s Family Advisory Council and iCAN. We anticipate the YLC will comprise 8–10 adolescents (12–17 years old) with an anxiety disorder. It will meet bimonthly to provide insight from the youth/patient perspective into “best practices” informed by their experiences and challenges with mental health services, and to provide essential guidance on study outcomes and potential barriers to enrollment, recruitment, and retention. We will integrate the YLC with iCAN and WeRise LA. WeRiseLA will also play an important role in dissemination of our findings, as we will work with our YLC to develop art projects that convey their experiences. We will explore other Community-Based Participatory Research methods (e.g., Photovoice [153, 154]) as vehicles for youth to express their experiences and perceptions as they inform our study. They will also help with interpretation and dissemination of study findings. We will convene the YLC bimonthly. Membership will include youth from all partner sites.

Resolving Conflict Conflicting opinions are inevitable in any sustained collaboration of diverse voices and perspectives, but when handled appropriately, it can have positive and desirable effects. We will employ community-based participatory research principles and tools to address conflict and differing opinions when they arise. We will have clear written rules for group interactions and decision-making. Advisory group members and the research team will be encouraged to recognize one another’s strengths and address any implicit academic or community stereotypes (e.g., “researchers only care about research”, “community members don’t have skills to bring to a research study”). Discussions will be facilitated by senior staff who have extensive experience conducting community-based participatory research with such advisory groups. We will identify an ombudsman from our standing SC CTSI’s Community Advisory Board to assist with conflict resolution, should intractable differences of opinions related to the study emerge.

### Conduct of the trial

#### Outcome measures

**Fig. 4:**
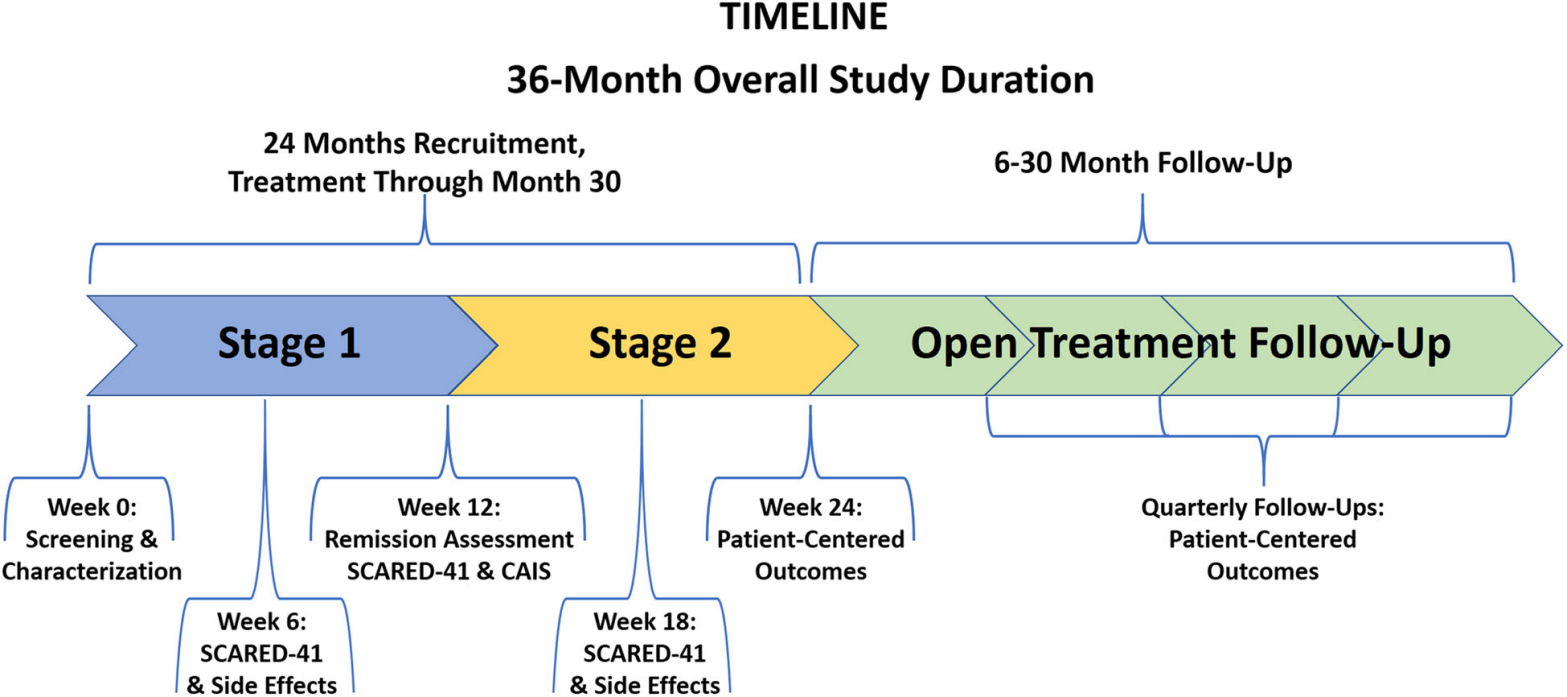
Participant Timeline for Assessments The overall study duration will be 36 months. Trial duration, subsuming stages 1 and 2 each 12 weeks long, is 6 months (24 weeks). Recruitment will occur in months 1–24, and treatment will continue through month 30. Naturalistic follow-up will continue after the 24-week trial to assess the durability of study treatment effects and will range from 6 to 30 months, depending on when participants were recruited into the study. Months 36–43 be devoted to data analysis and draft publication of the manuscript, with submission for publication by month 50

Active listening sessions with parents and youth with anxiety disorders conducted before study initiation discussed desired outcomes [155, 156] and the time requirements for assessments (< 75 min). Selection of our primary and secondary outcome measures were driven by these voiced preferences. Additional selection criteria included: documentation of the conceptual and measurement model; evidence for reliability and validity; interpretability of scores; validity; and change sensitivity [157]. Each measure will be obtained at baseline, week 12, and week 24 during the 24-week trial and quarterly during the ≥12-month follow-up. A subset of measures will also be obtained at week 6 and week 18 **(**Fig. 4**)**.

Primary Outcome Measure will be the patient ratings of anxiety symptom severity on the Youth SCARED-41 (Screen for Child Anxiety Related Disorders) [86, 136].

Secondary Outcome Measures will be the parent ratings of anxiety symptom severity on the Youth SCARED-41, and parent and youth ratings on the CAIS (Child Anxiety Impact Scale).

Outcome Measures for Exploratory Analyses are listed in Table 2**.** Many of these measures as listed in the Table were selected from our extensive listening sessions with parents and youth. Parents voiced wanting their child to be able to sleep through the night, manage change without anger or panic attacks, interact positively with peers and teachers, and attend school more consistently and without emotional outbursts. Desired outcomes for teenagers included feeling comfortable around peers, feeling confident in the classroom, feeling less worried about things outside their control, and being better able to manage emotions.

#### Baseline diagnostic assessments

Baseline assessments will generally be performed remotely via HIPAA-compliant Zoom, but they will be performed in person when parents and youth prefer.

##### Training of assessors

We will train staff on administration of the clinician-administered KSADS-COMP-Parent and Child [158] following instructions in the administration manuals. The KSADS-COMP is a computer-based version of the KSADS that is available in both English and Spanish. All our remaining assessments are either self- or parent-reports, or highly structured interview questions, that require minimal training to administer.

##### Parent-completed background materials

The *Patient/Subject Profile* is a systematic review of participants’ medical, psychiatric, and treatment history, as well as family history of psychiatric illnesses. SES will be quantified using the Hollingshead Index of Social Status [159], augmented with more contemporary measures of material hardship and perceived social status [160–162].

#### Randomization

**Fig. 5:**
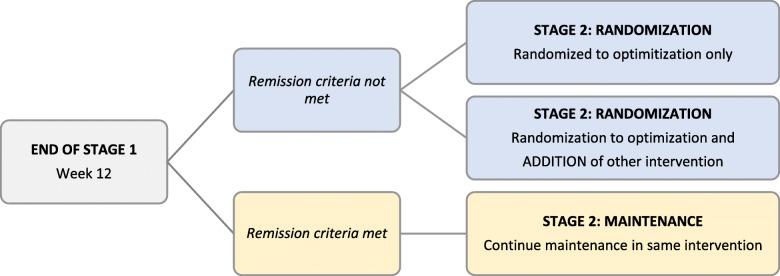
Schematic for Stage 2 Randomization At the end of Stage 1, participants who meet criteria for remission will continue in maintenance therapy with the same intervention as assigned in Stage 1. Those who do not meet criteria for remission will be randomized to either (1) optimization of the Stage 1 intervention they are already receiving, or (2) optimization of the Stage 1 intervention they are already receiving along with the addition of the other intervention (yielding combined medication + CBT)

In each randomization within trial Stages 1 & 2 **(**Fig. 5**)**, eligible and consenting/assenting participants will be randomized in a 1:1 allocation to the 2 treatment regimens. Randomization will be stratified by study site, age group [8–17], and baseline symptom severity (dichotomizing SCARED-41 scores as ≤33 or > 33, based on the median SCARED-41 score for participants having scores ≥25 in the CAMS [49] and LAMS [163] studies). Randomization will be further blocked, with a relatively small block size to ensure balanced randomization over the short term; block size will not be revealed to investigators or trial staff. The study statistician will develop and monitor fidelity to the randomization sequence. At the conclusion of week 12 of the Stage 1 intervention (medication vs CBT), anxiety symptoms and functional impairment will be reassessed. Participants who meet criteria for remission will continue maintenance-level therapy with the single-modality treatment received in Stage 1; non-remitting participants will complete Stage 2 randomization. The REDCap (Research Electronic Data Capture) randomization module will be used to randomize patients to study treatments [164, 165]; the study statistician will develop the stratified blocked randomization sequences. The randomization sequence will not be viewable. Randomization capability will be limited to the lead research coordinator and study statistician; randomization will only be available following confirmation that informed consent has been completed and all trial inclusion and exclusion criteria are met. Treatment assignment will be communicated from the lead research coordinator to the study coordinator who is assigned to screen, enroll, and administer assessments to that participant.

##### Maintaining treatment assignment

Clinicians and participants will be coached at study entry that adherence to the study treatment assignment is essential, as a relatively small proportion of crossovers can be detrimental to the trial. Medication adherence will be assessed with pill counts at each clinic visit.

##### Blinding & Minimizing Rater Bias

Because PCORI guidelines preclude paying for any component of clinical care, insurance will need to pay for study treatments, which in turn will preclude blinding patients and clinicians to treatment assignment. Nevertheless, all study assessments have been selected as parent- and youth-reports that require minimal to no interactions with research staff, thereby minimizing or eliminating rater bias from study staff. It is in this sense that we designate this study “single blind”.

#### Rationale for selection of fluoxetine

We selected the SSRI fluoxetine rather than an SNRI because SSRI therapeutic response is significantly greater and faster [21, 22, 129]. SSRI treatment effects begin to emerge within 2 weeks, sooner with higher doses [21], and approximately 50% of overall treatment-related improvement at week 12 occurs by week 4 [21]. Fluoxetine’s track record and safety are well established, and it is on nearly all formularies. When considering which SSRI to use in this study, we considered fluoxetine, sertraline, and escitalopram because they all have FDA-approved indications in pediatric patients. We also considered offering a choice of medication in the medication treatment arms. Our concern with this approach, however, even from a very limited set within a class, was that it could introduce a source of variance in response or treatment adherence that could be difficult to disentangle from the effects of treatment assignment within the SMART design. Therefore, we decided to constrain medication use to a single agent.

##### Paroxetine

Because of its risk of increased suicidality, which may be higher when compared to other SSRIs [166], and because it does not have any FDA-approved pediatric indications, we elected not to include paroxetine in this protocol.

##### Escitalopram

We did not select escitalopram because its efficacy as an antidepressant has not been demonstrated in patients under the age of 12 years [167, 168], and the likelihood of a similar suboptimal response in childhood anxiety seemed high. Moreover, extant data concerning escitalopram’s pharmacokinetics suggests optimizing its efficacy may require twice daily dosing [169]. Most importantly, we are aware of no prior studies of the use of escitalopram in the treatment of pediatric anxiety disorders.

##### Sertraline

Sertraline was not selected as the SSRI that we would use in this trial, for several reasons. First, it does not have proven antidepressant effects in pediatric patients and is not FDA-approved in this patient population for this indication, suggesting that its efficacy may be limited in treating the most common comorbid psychiatric illness in pediatric anxiety. In addition, like escitalopram, sertraline may require twice daily dosing for optimal use at lower doses [170], which can adversely affect treatment adherence and complicate prescribing by pediatricians who are new to prescribing psychotropics.

##### Fluoxetine

We selected fluoxetine as the SSRI to be studied in our SMART design for several reasons. First, it has more data than any other agent to support its safety and efficacy as a treatment for pediatric affective illnesses (especially depression). In addition, the long half-lives of fluoxetine and its active metabolite (norfluoxetine) allow it to be given as a once daily dose [171]. Once daily dosing, compared with twice daily dosing, has been shown to improve medication adherence in the treatment of chronic psychiatric illness [172]. Moreover, fluoxetine uniquely has evidence to suggest that increased dosages may benefit those who do not respond to lower doses [173], and therefore we are allowing flexible dosing for fluoxetine in this protocol. Finally, extensive discussions with leaders of our pediatric primary care network have suggested that the once-daily dosing, the wide range of doses over which fluoxetine administration is deemed safe in pediatric patients, and the simple upward titration in 10 mg increments will facilitate the training and comfort of pediatricians in prescribing medication in this study.

#### Administration of Fluoxetine

##### Training in fluoxetine administration

Prospective prescribers for the study will include child psychiatrists, developmental behavioral pediatricians, general pediatricians, and psychiatric nurse practitioners working in one of our study sites. They will undergo a 3-hour training for the study with two senior psychopharmacologists before being assigned any patients for treatment. Training will include an overview of study design, measures, inclusion and exclusion criteria, rationale for selection of fluoxetine as the study medication, and review of the pharmacology and drug interactions of fluoxetine, known side effects, assessment of side effects, reporting of adverse events, study dosing guidelines, remission criteria, and on-line training in the Columbia Suicide Severity Rating Scale.

Stage 1 Fluoxetine Dosing is flexible to maximize therapeutic effects while minimizing side effects, and based on literature for fluoxetine [174–176] and FDA regulatory approvals [177]. The study’s starting dose, and minimum permitted, is 10 mg/day; should that not be tolerated, the patient will be withdrawn from active treatment (but not from study follow-up). After 1 week at 10 mg/day, the dose will increase to 20 mg/day. After completion of week 4, 10 mg/day dose increases are permitted every other week as tolerated, up to a maximum daily dose of 80 mg/day. If the patient is not in remission and does not have dose-limiting side effects, written guidelines will encourage the prescribing physician to increase the dose of medication. If patients are on doses > 20 mg/day, the total daily dose can be prescribed either once daily or split into twice daily administrations. If dose-limiting side effects occur, dosages can be reduced by 10–20 mg/day. Patients can be prescribed a dose that previously was not adequately tolerated if: 1) at least 2 weeks have elapsed since the dose was reduced, and 2) the patient/parent agrees to re-try the higher dose.

Stage 2 Fluoxetine Dosing Most studies included in the meta-analysis showing that most improvements occurred in the first 6–8 weeks of SSRI therapy used fixed medication dosages, suggesting that further response optimization can be achieved with upward dose titration in Stage 2, when the initial treatment in Stage 1 does not achieve either maximum medication dosing or clinical remission. Approximately 40% of youth with pediatric anxiety disorders fail to respond to either SSRI or CBT [21–23]; rates of failure to achieve remission are even higher (60–80%) [21, 22, 24–27, 30], and rates of relapse are very high (approximately 60%) [16, 28, 29], particularly in those who have more residual symptoms and functional impairment following acute treatment [29]. Therefore, the goal of achieving clinical remission is imperative in improving outcomes, which requires assertive optimization of medication dosing.

Per the above protocol, the fastest that patients can achieve a maximum dose of 80 mg will be week 15 of the study. In every pediatric psychopharmacology study published thus far that has used a flexible dosing schedule, however, the average medication dosages achieved have been considerably lower than the maximum dose allowed. Therefore, participants will not reach the maximum dose in the 12 weeks of Stage 1 of our SMART design. In addition, upward titrations will be slowed in some youth due to the emergence of side effects or because clinicians will be reluctant to increase the dose only 2 weeks after the last dose increase, instead wanting more time to observe for clinical improvement. The additional time in Stage 2 will allow higher overall doses to be achieved.

Therefore, optimization procedures in Stage 2 will be identical to those of Stage 1, though clinicians will be encouraged to make every possible effort to achieve the maximum dose of 80 mg unless or until remission is achieved. We will allow upward-titration in the presence of an inadequate clinical response, at evidence-based intervals; similarly, we will allow downward titration should dose-limiting side effects occur. Using these strategies, fluoxetine treatment will be optimized based on extant scientific literature in our pediatric population. Medication adherence will be monitored via pill counts at each clinical visit.

We will closely monitor medication dosing and patient tolerance during the first two cases assigned to each prescribing physician. Monitoring will be made possible through dosing and side effect data that a study coordinator will extract from the patient’s electronic medical record and enter into the study’s REDCap database. If the patient is tolerating the medication well and is still symptomatic, prescribers will be encouraged to increase medication dosage according to the written study guidelines. After completion of these first two cases, senior study psychopharmacologists will be available to prescribers for brief email or phone consultation if prescribers have questions about dosing or side effect management. This model of initial oversight in two cases and subsequent availability for brief consultation is intended to mirror the training and subsequent consultation that will be provided in the CBT treatment arms. It also adheres closely to real-world practice in psychopharmacology, particularly for general pediatricians.

Rationale for 12-Week Duration of Medication Study Stages and Evidence for the Benefit of Medication Optimization The vast majority of RCTs for medication therapy of anxiety and depression have been 12 weeks or less in duration [20]. For this reason, only a modest evidence-base exists on treatment beyond 12 weeks. For example, the duration of CAMS trial, which compared CBT with medication and the combination, was only 12 weeks long. Indeed, we have been able to identify only 2 RCTs using either an SSRI or SNRI for the treatment of pediatric anxiety that were longer than 12 weeks in duration (both were 16 weeks) [178, 179]: in both the SSRI (paroxetine) [178] and SNRI (venlafaxine) [179] studies, mean therapeutic response did not improve from treatment week 12 to 16.

Furthermore, a meta-analysis has shown that the effects of SSRIs begin to emerge within 2 weeks of initiating treatment, and sooner with higher doses [21]. Approximately 50% of overall treatment-related improvement observed at week 12 occurs by week 4 [21], suggesting that the remaining 50% of improvement occurs over the last 8 weeks of treatment, approaching asymptote by week 12. Most of the studies included in the meta-analysis used fixed dosages. However, using fixed doses also suggests that further optimization of response can be achieved with upward dose titration in Stage 2, when the initial treatment in Stage 1 does not achieve either maximum medication dosing or clinical remission, and remission is what we are hoping to achieve with the optimization of medication therapy in Stage 2 of our SMART design. The goal of achieving clinical remission is imperative in improving outcomes, which requires assertive optimization of medication dosing.

#### CBT stage 1 implementation

We will use the *Coping Cat* program as the behavioral intervention for this study. *Coping Cat* is an established evidence-based CBT treatment for pediatric anxiety [180–182] that has been studied rigorously for more than 25 years. The CBT strategies that form the core of *Coping Cat* have been subjected to years of extensive research through clinical trials [183, 184], have been shown to be highly efficacious in addressing symptoms of a range of anxiety disorders, and are widely available at low cost. It is delivered in individual therapy sessions with anxious children.

*Coping Cat* comprises 4 core functional components **(**Table 1**)**:

1. Building a Collaborative Working Alliance: recognizing and understanding the emotional and physical reactions to anxiety;
2. Undergoing exposure without Avoidance: clarifying thoughts and feelings in anxiety-provoking situations;
3. Developing Coping Efficacy: developing plans for effective coping (e.g., modify anxious self-talk into coping self-talk, or determine what coping actions might be effective);
4. Engaging in Reward: evaluating performance and giving self-reinforcement.

The Coping Cat workbook is used for children aged 8 to 13 years, and the parallel C.A.T. Project workbook is used for ages 14 to 17. For both age groups, the sequence of sessions follows the same structure: Introduction, including psychoeducation and development of an individualized anxiety hierarchy; Skills Building, including relaxation training and coping skills; and Experiential Practice, including exposure and practice of coping skills, moving from the least to most anxiety-provoking situations from an individualized anxiety hierarchy. *Coping Cat* will be delivered over 12 weekly therapy sessions in Stage 1 of our SMART study with minimal modification to the original *Coping Cat* protocol.

*Coping Cat* therapists will be those who normally provide therapy in the study sites; these include licensed mental health professionals (e.g., psychologists, social workers) and trainees (e.g., psychology interns, fellows). All will undergo *Coping Cat* training and participate in ongoing consultation and fidelity measurement. To promote optimal representativeness and external validity, there will be no requirements for prior training; *Coping Cat* training has demonstrated effectiveness with clinicians ranging from graduate students [180, 181] to experienced psychotherapists [49], with strong treatment effects in each case.

##### Adaptations to *Coping Cat*

We have included very few planned adaptations to the form of *Coping Cat* administration in this trial. In Stage 1, clinicians will deliver 12 sessions of *Coping Cat* per the manualized protocol. These 12 sessions will be condensed from the original 16 *Coping Cat* sessions by consolidating the psychoeducation and skills building sessions, adaptations that have been designed in consultation with the developer for *Coping Cat*. An additional, minor planned adaptation will be that the “final” session 12 activities will not be framed as termination, but rather as taking stock of progress, review of skills learned, and preparation for Phase 2. CBT optimization in Phase 2, will involve more extensive adaptations to the *Coping Cat* protocol and will reflect an intensification of the CBT maintenance treatment protocol from the CAMS trial. In the post-acute phase of CAMS, patients in the CBT arm were offered maintenance treatment “designed to reflect the manner in which the active CAMS treatments most appropriately be delivered in clinical settings.” This maintenance treatment consisted of 6 additional CBT sessions to be delivered over 6 months, focusing on additional exposure practice and coaching in the application CBT skills to emerging life stressors [42]. The content of these CAMS maintenance sessions directly reflects the content we have incorporated into our Stage 2 CBT optimization phase, namely, continued intensive exposure practice and skills review. Our planned adaptation is designed to *intensify* the maintenance protocol from CAMS by increasing the total number of sessions (from 6 to 12) and the density of delivery (weekly versus monthly) and thus to optimize the dose of exposure learning.

In addition to our two planned adaptations to the *Coping Cat* protocol, unplanned adaptations may occur across arms. Unplanned adaptations may include deviations to address crises that emerge (e.g., case management, suicide risk assessment) and necessary adaptations to content to meet developmental or cultural diversity needs, among others. Any deviations from the manualized structure and content of *Coping Cat* will be recorded via a session adherence form completed by the clinician and presented/discussed at study team meetings. The CBT leadership team will decide how to address and formally record adaptations for the purposes of study adherence measurement, data analyses, and process evaluation, and will also provide feedback to CBT clinicians as necessary to prevent unnecessary deviations from the treatment protocol. Note that even within efficacy trials for CBT, crisis management sessions are built into the design of the protocol, as they represent good clinical care, compliance with legal requirements (e.g., mandated reporting), and appropriate adherence to evidence-based treatment [42].

##### Risk of early termination of CBT treatment

Many interventions for internalizing disorders do not have immediate effects; indeed, families are routinely told that symptom improvement is unlikely during the first several weeks of SSRI treatment. Similarly, as part of the CBT *Coping Cat* intervention, patients and families are provided psychoeducation at the beginning of the protocol describing CBT as a skill-building intervention that will work through repeated practice. As such, the CBT model also does not prime patients or families to expect immediate improvement. Nevertheless, in a relevant prior study [49], 139 children were randomized to 12 weeks of *Coping Cat* treatment, and none of those children withdrew from treatment during the entire 12 weeks (though 6 (4.3%) were lost to the study). This suggests that the risk of early withdrawal from CBT is low and that the number will likely be small. Moreover, these prior findings mirror service utilization data from our treatment sites.

#### CBT stage 2 implementation

For those participants randomly assigned to continued/intensified CBT for Stage 2, weekly CBT will continue for an additional 12 weeks. The protocol for CBT optimization is a planned adaptation of the CBT maintenance treatment delivered in the landmark CAMS anxiety efficacy trial [49]. In the post-acute phase of CAMS, patients in the CBT arm were offered 6 additional CBT sessions to be delivered over 6 months, focusing on additional exposure practice and coaching to maintain application of CBT skills in the face of emerging life stressors [42]. The *content* of these CAMS maintenance sessions directly reflects the *content* we have included in our Stage 2 CBT optimization phase, namely, exposure and skills practice. However, our planned adaptation is designed to *intensify* the maintenance protocol from CAMS by increasing the total number of sessions (from 6 to 12) and the frequency of delivery (weekly versus monthly). Furthermore, the goal of the 12 sessions of optimization is not simply to maintain gains from the end of Stage 1, but rather to produce substantial additional clinical change in Stage 2. As such, exposure practice in Stage 2 will consist of intensification of practice exercises, moving up the patient’s hierarchy to tasks of increasing difficulty, and promoting patient mastery. It will also involve consolidation and review of previously learned CBT coping skills. This approach corresponds to what would normally occur in clinical practice if a patient did not fully respond to the initial acute phase of *Coping Cat*.

No new CBT techniques will be introduced in Phase 2. This content directly parallels the content of CBT maintenance treatment from CAMS, but the Phase 2 optimization sessions in this trial are delivered at a higher dose and density than the maintenance sessions prescribed in CAMS. This planned adaptation is designed to optimize response in our sample of diverse, underserved and clinically complex youths through the mechanism of increasing the dose of exposure learning. This approach corresponds to what would normally occur in clinical practice if a patient did not fully respond to the initial acute phase of *Coping Cat*.

##### Rationale and evidence for the benefit of CBT optimization in stage 2

The rationale for our optimization protocol comes from three sources. First, the content and general structure of the sessions are drawn from the CBT maintenance protocol of the gold-standard CAMS anxiety trial [42]. In CAMS, this was “designed to reflect how the … treatment would be delivered in clinical settings” over an extended care time frame and focused on exposure practice and coping skills review, without the addition of other new material [49]. Thus, additional enactive ***practice*** was emphasized as the major clinically relevant task for optimizing CBT in our protocol. Second, analyses of process data from the CAMS trial suggest that quality of exposures may be a central element in therapeutic change for anxious youths in CBT. Amount of time spent in session on exposures, focus on mastery of difficult exposures, and child adherence with tasks and mastery of skills -- all these elements of treatment delivery predicted better clinical outcomes in the CAMS trial [119]. Our CBT optimization protocol thus focuses on increasing the dose and ***intensity of exposure learning*** for youths. Third and finally, our focus on practice and intensity was informed by previous findings suggesting that disadvantaged youth may have lower engagement in key tasks of CBT and may have less mastery of material, leading to poorer outcomes. In CAMS, Black/African-American youths demonstrated lower attendance at CBT and medication management sessions, and they were rated by therapists as exhibiting less involvement and compliance with treatment. Perhaps as a consequence, they also showed a lower level of mastery of CBT concepts [41]. Statistical control for these process factors and SES eliminated racial differences in outcome. Similarly, patient non-adherence (poor attendance, low homework completion, poor compliance in session) was associated with number of parents present in the home (with the best outcomes for two-parent families), although indices of non-adherence varied in their power to predict clinical outcomes [124]. Our SMART design is intended to fill major gaps in the evidence base on the sequencing and optimization of CBT and medication treatment and, critically, to do so in an underserved population. Given these process-outcome findings related to engagement, we sought to level the playing field for disadvantaged youths by having our optimization protocol focus on mastery of skills as the goal of Stage 2 CBT and to provide an extended set of sessions on this topic in order to increase the likelihood that all youths will receive a high-dose of care.

##### Addressing non-responders’ willingness to continue CBT in stage 2

Relevant data come from the analysis of CAMS outcomes at week 24 and 36 [42]. In Phase 1 of the CAMS anxiety trial, youths were randomized with either CBT, SSRI, Combination (COMB), or placebo and clinical outcomes assessed at 12 weeks. After this acute phase, in Phase 2, responders were offered 6 months of continuing monthly maintenance treatment in their originally assigned condition. Non-responders in the treatment arms were referred to community providers for general outpatient treatment, and non-responders to the placebo condition were offered their choice of active study interventions. During Phase 2, outcomes were assessed at week 24 and 36, mapping onto our post-intervention assessment for the second half of our SMART study. In the CAMS sample, youths had excellent retention over this follow-up period, with nearly 80% of youths completing study assessments. Of note, the CAMS authors did not report evidence of differential study attrition by assigned study condition, participation in maintenance treatment, or responder status. Thus, non-responder youth assigned to continued CBT were just as likely to remain in treatment (with high rates of retention) as youth assigned to a new, active intervention. These findings suggest good acceptability of clinical recommendations for the treatment path following an acute phase of intervention.

#### Process evaluation

Our planned process evaluation will use quantitative and qualitative methods to assess treatment fidelity and related attributes (e.g., dose), and to study and assess the hypothesized causal pathways leading from our interventions to patient outcomes **(**Fig. 1**)**. We describe here details of our approach to fidelity assessment and planned mediation analyses. We will supplement these with qualitative process evaluation activities using data from interviews and surveys.

##### Treatment modality

Process evaluation of treatment modality in our causal pathways will focus on evaluating the fidelity to medication and CBT treatment. Medication treatment fidelity will be measured by sessions attended and pill counts at each session. CBT treatment fidelity will be measured by session attendance and assessing the extent to which the 4 core functions of *Coping Cat* CBT are accomplished. Fidelity to the “forms” will be measured via specific *Coping Cat* fidelity measurement activities (below). We will also assess the youth’s acquisition of each core function of the program (i.e. alliance-building, skills building, exposure, reward) via objective measurement as well as qualitative assessment. The assessment of both function and form, and subsequent comparison of the relation between core functions and the forms used to achieve them will enable the study to inform whether the core functions of the treatment are achievable via various alternative pathways (e.g., forms).

##### CBT training and quality assurance

CBT training and consultation will involve an initial 2-day training workshop for all study therapists. The first day of training will cover the theoretical foundation and foundational skills for CBT for anxiety disorders. Then training will move to the specific structure and content of the *Coping Cat* (including C.A.T.) program and guide clinicians through the manual in detail, using case examples, video, and trainer modeling. The second day of training will involve extensive education and training in exposure, including theoretical background, practical implementation, problem-solving barriers, and modeling/role play by trainers as well as role play opportunities and feedback to participants. The training will also briefly summarize the evidence-base for *Coping Cat*, as well as review in-depth our study design and quality control procedures.

Once clinicians have completed the initial training workshop, they will be assigned to a consultation group with an experienced CBT clinician. Our training plan reflects real world practice in that we do not require portfolio submission and fidelity-rated recordings in order to “graduate” as a *Coping Cat* therapist. Instead, clinicians will be required to receive weekly consultation on their first two cases, and record their sessions for retrospective fidelity measurement. CBT trainers will be available throughout the study to CBT therapists for questions they may have in implementing *Coping Cat* with subsequent patients. If clinicians are trained in both Coping Cat CBT and fluoxetine administration, their caseloads will be balanced with patients from each arm of the study to control for any possible clinician-level effects.

##### CBT fidelity measurement

All CBT therapists will be provided audio recording devices and asked to audio-record their sessions. We will randomly select 1 study case from each clinician (assigned after their initial 2 training cases); a trained fidelity rater will listen to all 12 sessions of this case and rate fidelity using a standardized fidelity checklist developed for *Coping Cat* [180, 181]. These fidelity rating checklists will allow raters to record the delivery of treatment components during each session and calculation of percent fidelity to the treatment model for each CBT therapist and overall, across all therapists in the study. We will also include a measure of general therapy competence that has been used with *Coping Cat* and can help assess the quality of treatment delivery, including exposure practices [185].

With the *Coping Cat* fidelity checklist used to measure clinician adherence to *Coping Cat*, and required record-keeping for number of sessions completed, it will be possible to conduct analyses of the association between *Coping Cat* adherence and dose, on the one hand, and improvement on patient centered outcome measures of anxiety symptoms on the other. In addition, given the central role of exposure and tolerance of negative affect in the *Coping Cat* conceptual model, and following procedures used in the CAMS study [119], we will have therapists complete brief reports following each session in which they rate the child’s overall adherence with treatment procedures, mastery of the information/skill covered in the session, and they will provide 3 kinds of information related to exposures: (1) the number of exposures used in the session, (2) difficulty level of the exposures (based on the child’s ‘subjective units of distress’ ratings), (3) level of skill/mastery shown by the child during the exposures. This will permit us to develop an index of tolerance of exposure and exposure mastery shown by the child, on the one hand, and patient-centered outcomes (Table 2) on the other.

#### Long-term follow-up

Recruitment will occur over the first 2 years. The last patients recruited will complete the 24-month trial at 2.5 years after study initiation, leaving a minimum of 12 months follow-up duration for those recruited last. We will continue following patients recruited earlier to provide longer-term follow-up data. Assessments will occur quarterly and will be identical in content to those obtained during the 24-week trial. During follow-up, patients and their clinicians will be able to select whatever treatment they wish, and at whatever frequency and intensity deemed desirable; insufficient responders or those who relapse during the follow-up period may switch to another medication or psychosocial therapy at the treating clinician’s discretion. We will encourage clinicians and patients to continue with successful treatments begun during the trial, however. Booster CBT sessions will be provided as needed, as occurs in regular (good) clinical practice. These sessions will not be scheduled at regular intervals but will instead occur in response to patient need and according to the provider’s clinical judgement. All booster sessions will involve review of the youth's FEAR plan developed during the initial phase of treatment and will use this plan as a base to address residual or recurring issues, avoidance, and functional impairment. The number of booster sessions, other treatments delivered, and any changes (including discontinuations), will be tracked and recorded carefully for consideration in analyses.

#### Adverse events (AEs)

These will be elicited by a combination of structured questions and direct, open-ended inquiry of patients and parents in each treatment arm of the study using the Pediatric Side Effect Questionnaire (a modified version of the Antidepressant Side Effect Questionnaire) at weeks 6, 12, 18, and 24 of the study. Clinicians will be requested to complete this form at each clinical visit; the form will be placed in the clinic chart, which a coordinator will then extract and enter into the study’s REDCap database. We will use the FDA definition to define a serious or unexpected AE [186]; for each reported AE, we will document whether it meets regulatory criteria for a serious or unexpected AE, and the presumed relationship to study procedures. Federal guidelines and recommendations for reporting serious or unexpected AEs to site IRBs and study’s DSMB will be followed [186]. Height, weight, blood pressure, and pulse will be assessed at each in-person study visit, and we will request that parents measure all except blood pressure for each telehealth visit [177]. The development of suicidality during treatment is a possibility; participants will therefore be assessed at each study visit with the Columbia-Suicide Severity Rating Scale (C-SSRS) [104]. Patients will be withdrawn from the study if continued participation is deemed unjustified due to risk of self-harm, and each study site will take appropriate measures to ensure safety.

#### In-person and telehealth treatment

Reflecting the values of real-world implementation at the heart of PCORI’s mission, we will allow in our study the delivery of care through either in-person visits or telehealth, as routinely conducted at each of our performance sites. This modification is motivated in part by the conversion of all mental health care across our sites to telehealth to comply with safety considerations during the COVID-19 pandemic. In addition, several of our performance sites, even before the pandemic, have long histories of conducting pharmacotherapy entirely by telehealth, given the limited child psychiatry support available to cover wide geographic service regions. Moreover, the provision of psychotherapy by telehealth was accelerating at most of our sites before the pandemic. All our sites at the time of study initiation are providing all non-emergency mental health care in this medium. Following resolution of the pandemic, we anticipate that delivery of care in our study will include both telehealth and care that is delivered face-to-face. We will document for each treatment session whether it was conducted in person or via telehealth, whether the patient’s video platform was a computer or a smartphone, and whether any technical difficulties in implementation were encountered.

For patients receiving treatment via telehealth, we will assess the acceptability of telehealth treatment delivery using structured questionnaires administered to patients, parents, and clinicians after weeks 1 and 12 in Stage 1 of the study. These structured questions have been adapted from questions found in a detailed review of past studies of acceptability and feasibility for telehealth treatment delivery to either youth or adults [187–194], as well as from our initial meetings discussing telehealth experiences with patient, parent, and clinician stakeholders. We will also conduct a process evaluation using exit interviews of each of these 3 individuals (patient, parent, clinician) after week 12 using open-ended questions. This combination of structured and open-ended inquiries will provide a rich and comprehensive understanding of patient, parent, and clinician experiences with telehealth in our study.

#### Methods to prevent and monitor missing data

We will use several strategies to prevent and reduce missing data, particularly trial outcome measures. We will use REDCap [165] for data collection and entry. REDCap is a secure, HIPAA-compliant, widely used web-based research application that supports calculations and branching logic programming. It provides (1) an intuitive interface for validated data entry, (2) audit trails for tracking data manipulation and export, (3) automated export procedures for seamless data downloads to common statistical packages, and (4) tools for importing data from external sources. We will program our REDCap database to require responses to all survey and interview questions and real-time documentation of reasons for missing data. Reasons for truly missing data (e.g., if participants are uncomfortable completing specific items) will be completed at the time of data collection and documented during data entry. This will also reduce missing data due to data entry errors. The trial data management protocol will include immediate post-data entry notifications, tracking of rectifications of missing data, and daily review of new data entries with immediate notification to data entry personnel and site coordinator for rectifications needed for missing data. We will attempt to complete trial outcomes at protocol-specified assessment times on all randomized persons, even those who have dropped from the study. Our project analyst will review data weekly to ensure completeness; a weekly report to investigators and study coordinators will identify outstanding missing data that needs to be completed, as well as a time field indicating number of days since the missing data query was first generated. The study protocol will provide instructions for use of these strategies and procedures.

#### Data management

We will employ a detailed data management plan to ensure the integrity and accessibility of study data. We will store all data on secure servers in an integrated REDCap database [164, 165]. All clinical, behavioral, and demographic information for each participant will be entered into the database. We will use REDCap to create a web-based data entry interface, perform Stage 1 and Stage 2 randomizations, update participant information, manage baseline and follow-up visits, and track participant status, and to export data efficiently in various self-documenting forms directly into SPSS, SAS, R, or Excel using REDCap’s de-identification option, thereby ensuring that exported data are complete, self-explanatory, and de-identified. We will also design and implement a database system to acquire and integrate all paper and electronic files from participant assessments. We will implement procedures to maintain data integrity, reliability, security, and accessibility, ensuring that all data are consistent with HIPPA and other federal regulations and with applicable policies of our local IRBs. We will ensure that all participant data have been transmitted and incorporated into the central database, and fully documented, in a timely manner. We will also manage and document data requests, make data available to investigators through a Data Use Agreement, and maintain archives of all analysis datasets and datasets provided to other investigators.

##### Tracking system

The study's REDCap database will aid study coordinators and participating sites in tracking participant visits and data collection. The password-protected, structured web-based portal will include real-time reports to identify participants who are due for a follow-up visit, list the type of data to collect in that visit, and list any information that has been missing from past visits. All data modifications will be logged to maintain accountability for all entries and edits. Automatic monthly emails will be sent to investigators and coordinators alerting them to upcoming visits of their participants.

##### Security

We will ensure the database systems, data access policies, and data transmission protocols exceed HIPPA regulations and data security standards for IRBs. Only designated staff will have direct access to the database. The front-end web portal to the database will be accessible to all investigators and study coordinators. The REDCap server is housed behind multiple firewalls in a locked and guarded USC data center equipped with security cameras and intrusion detection systems that is staffed by security at all times. All electronic connections to the REDCap environment are encrypted. Only system administrators at the data center are authorized to access the back-end database server directly by logging into a virtual private network. We will institute policies that (a) temporarily deactivate user accounts that have not logged into the system within a specified time; (b) automatically require staff to change passwords at regular intervals; (c) match current users against current study staff, and terminate user accounts for staff who no longer are associated with the project; and (d) institute independent audits at regular intervals by our ISO to assess HIPPA compliance and security. All data files transmitted to investigators will be encrypted and password-protected at the highest level of data encryption, then transmitted via a secure File Transfer Protocol. The password used to encrypt the file will be transmitted separately from the file.

### Sample size and power

SMART designs are commonly but incorrectly assumed to require prohibitively large sample sizes [54]. It is common to think, for example, that data from a study design such as ours will be analyzed in a 6-way ANCOVA that compares outcomes across all 6 subgroups, which would indeed likely require a large sample size. That analysis, however, does not correspond with our primary aims and hypotheses, which are tested as two main effects and their interaction, and which require a sample size considerably smaller than for a 6-way ANCOVA [54]. We have made it our priority to identify a sample size that will allow us to test Stage 2 effects with sufficient power. We obtained the estimates of effect size (ES, in SD units) required for estimating our required sample size as follows:

(1) We used treatment ESs from the CAMS efficacy trial, which compared combined medication+CBT to monotherapies (medication alone, CBT alone) and placebo. CAMS reported quantitative anxiety outcomes using the Pediatric Anxiety Rating (PARS) scale. Outcomes did not differ significantly in CBT alone vs medication alone (though group mean differences on the PARS slightly favored medication over CBT). However, CAMS did find large ESs for combined medication+CBT compared to monotherapy, with a somewhat larger difference compared to CBT alone than medication alone.

(2) Using augmented CBT in CBT non-responders as the base, relative ESs reported at 24 weeks in CAMS were used as ES estimates for CBT + medication (i.e., medication added to CBT), medication alone, and medication+CBT (i.e., CBT added to medication). For Stage 1 remitters, we used an ES of 0.2 above their non-remitter counterpart.

(3) Group main effect ESs were then computed for a Stage 1 remission rate of 40%. Our estimated Stage 1 remission rate of 40% is a conservative estimate based on the CAMS remission rate of 35% achieved for ethnic minorities, when applying remission criteria less stringent than ours [30]. In CAMS, the 12-week remission rate was also approximately 10% higher in medication-only compared to CBT-only. We used this 10% difference in these calculations to compute ESs and resulting sample size estimates. For Main Effect 1 (start with medication vs start with CBT), ESs for all 3 medication or CBT groups (1: remitter; 2: non-remitter➔optimize monotherapy; 3: non-remitter➔medication+CBT) were computed as a weighted average of the group-specific ESs. Weights were the remission rate, with the non-remitter weight (1 minus remission-rate) split equally among the 2 randomized non-remitter groups (reflecting 1:1 Stage 2 randomization among non-remitters). For Main Effect 2 (among Stage 1 non-remitters, optimize Stage 1 treatment vs add the other treatment), ESs were computed as a weighted average of the ESs for monotherapy optimizers (medication+, CBT+) and a weighted average of the combined (medication+CBT, CBT + medication) groups. These weights were again 0.5*(1- remission-rate).

[4] We then computed the sample size required to detect Main Effect 2 (optimize monotherapy vs add the other therapy in non-remitters) at 80% power and tested at a 2-sided alpha = 0.05. From our computations above, the Main Effect 2 ES was approximately 0.40 SD, requiring a sample size of 194 participants among Stage 1 non-remitters. Increasing the sample size of 194 by the non-remission rate (N divided by non-remission rate) and by the estimated 20% dropout rate, yielded a sample size of 404 (202 per group) for Stage 1 remission rates of 40%. Randomizing 404 participants, with an estimated 324 finishing the trial and a remission rate of 40%, will provide the ability to detect an overall Main Effect 1 (medication first versus CBT first) ES of ≥0.31 SD with 80% power.

#### Power analyses for telehealth-based treatment delivery

The above effect size estimates used the CAMS 24-week trial results to estimate comparative effect sizes for the 6 groups (remitters, non-remitters augment Stage 1 intervention; non-remitters add intervention). Two RCTs provide data to estimate effect size estimates for treatment delivery via telehealth. The first [195] is a meta-analysis of 26 RCTs comparing videoconferencing-based telehealth vs in-person delivery of psychiatric services, which included both pharmacotherapy and psychotherapy. The summary effect size (telehealth vs in-person) = − 0.11 (95% CI -0.41, 0.18), a non-significant difference with the direction of effect favoring tele-health delivery. The second [196] is an RCT comparing telehealth vs in-person CBT over 24 weeks (the same duration as our study) in 115 youth with adolescent anxiety disorder. For consistency with our sample size calculations (which used the primary outcome from the CAMS trial, the clinician-rated Pediatric Anxiety Rating Scale), we estimated the effect size for the Clinician Severity Rating outcome measure, which was 0.05 (95% CI -0.77, 0.95). This is a non-significant difference, with the direction of effect favoring in-person CBT. We used these estimated in-person vs telehealth effect sizes to estimate adjustments to our original effect sizes, adding 0.11 to all of the medication group effects (medication remitter, medication non-remitter: augment, medication non-remitter add CBT), and subtracting 0.05 from all the CBT group effects (CBT remitter, CBT non-remitter: augment, CBT non-remitter: add medication). We concluded that there is no effect on the overall main effect 2 effect size used to estimate sample size, because the main effect 2 comparison is: Augment (Medication+; CBT+) vs Add (Medication, add CBT; CBT, add Medication), and the adjusted remote effects appear in both Augment and Add groups, so these effects cancel out. The same conclusion is obtained if we separately consider ONLY a remote medication effect, or ONLY a CBT effect.

### Data analysis plans

Upon completion of the final trial participant, we will complete a final database review and finalize data queries, then lock the trial database for final analysis. To evaluate baseline comparability, we will compare demographic and clinical characteristics, and trial outcome measures, at baseline between treatment groups. Continuous baseline measures will be summarized by mean (SD) or median (IQR), and categorical measures by frequency (percent). Standardized group differences will be computed and presented for all baseline variables.

We will conduct an intent-to-treat analysis, by which subjects will be analyzed according to randomized intervention, consistent with standard practice in clinical trials. Distributions of outcome variables will be graphically examined; normalizing transformations will be applied if needed. The 24-week continuous measures of primary and secondary trial outcomes will be compared between treatment groups using general linear models, with the 24-week measure as the dependent variable. SMART design groups will include Stage 1 randomized group and Stage 2 randomized group. The randomization stratification factors (age group,clinical site, and dichotomized baseline parent SCARED-41), as well as baseline measures of the trial outcome (Youth SCARED ratings), will be included as model covariates. Results will be summarized by treatment group means (SDs) and mean treatment group differences (and 95% confidence intervals). No interim analyses are planned.

To **test Main Effect 1**, a 2-group (Stage 1 randomization) analysis of covariance will be used, comparing subjects randomized to medication first to those randomized to CBT first [53, 54, 197]. To **test Main Effect 2** among Stage 1 non-remitters, a 2-group analysis of covariance will also be used, comparing (a) non-remitting subjects randomized at Stage 2 to optimization of their Stage 1 monotherapy, vs (b) non-remitting subjects randomized at Stage 2 to combination treatment (medication+CBT or CBT + medication) across levels of Stage 1 randomization. Finally, to test whether one sequence of treatment modalities (CBT➔CBT; CBT➔med; med➔med; med➔CBT) is significantly better or worse than predicted from the two main effects, an interaction term of Stage 1 randomization with Stage 2 randomization will be added to the model. The addition of the interaction term will allow estimation and testing of each of the 4 treatment sequences [53, 54, 197].

To reflect the SMART design, two additional analytic issues must be addressed. First, the comparison of 4 sequences requires replication of the Stage 1 remitters, to reflect these subjects’ contributions to both the Stage 2 augmentation and combination treatment strategies (i.e., each Stage 1 remitter contributes 2 observations to this analysis). Second, to reflect the fact that Stage 1 remitters are only randomized once, whereas Stage 1 non-remitters are randomized twice, regression weights will be used, such that each subject is weighted by the inverse probability of ending up in the sequence to which they were randomized (weight of 2 for remitters, 4 for non-remitters). As the Stage 1 remitters contribute two observations to these analyses (and thus correlated outcomes) in comparing treatment sequences, a sandwich-based variance estimator will be used to obtain robust standard errors for effect estimates [53, 54].

#### Durability of the 24-week intervention

This will be assessed using the repeatedly-measured outcomes collected quarterly for 12 months following end of the trial intervention, following the theoretical and simulation approaches and results provided by Lu et al. (2016) [198] and Li (2017) [199]. These data will be modeled using marginal means models with generalized estimating equations, with weighting and replication of observations based on stage 1 responder status, as recommended for SMART designs. Using this approach, the post-intervention linear slopes as well as end-of-study absolute values in trial outcomes will be compared among the adaptive treatment strategies (assessing group-by-time interaction terms); we will also consider possible non-linearities in post-intervention trajectories with addition of polynomial terms for time of assessment. Adverse events will be categorized using the MedDRA coding system and compared between treatment groups using exact methods for comparisons of proportions.

#### Multiplicity

The primary outcome (Youth SCARED) will be tested at an α = 0.05. Evaluation of secondary, exploratory, and all subgroup analyses will control for the false discovery rate [200].

### Sensitivity analyses

These will assess the impact of analytic and modelling assumptions related to selection of covariates, handling of missing data, and adherence to randomized intervention. They will compare parameter estimates and statistical conclusions of treatment group differences on outcomes to study the differential impacts of various assumptions, and will include: (1) additional baseline covariates that differ between groups, evidenced by a standardized difference ≥ 0.1; (2) trial dropouts in the 24-week analysis, using multiple imputations to attain 20 complete trial datasets and summarizing treatment effects over the repeatedly imputed datasets; (3) an adherence-based analysis, limiting analyses to subjects who participated in ≥80% of planned intervention contacts and took ≥80% of medication doses based on pill counts..

#### Effects of treatment modality

We will evaluate the association of percentage telehealth sessions with session attendance and completion of the trial. We will also assess the influence of telehealth delivery variables (whether sessions were conducted in person or via telehealth, whether the patient’s video platform was a computer or a smartphone, and whether any technical difficulties in implementation were encountered) on treatment outcomes by including them as covariates in our sensitivity analyses.

### Statistical methods to address missing data

Trial dropouts will be reported by follow-up visit and summarized by reason for dropout. Missing data for trial outcomes will be analyzed to provide insight on the missing data mechanism. We will inspect patterns of missing data by each randomized group. Baseline characteristics will be compared between participants with and without complete outcome data. Participant characteristics found to be related to missingness will be correlated with values of the outcome variable (baseline values and among participants who have complete data); we will evaluate the relationship of baseline values of the outcome variable to missingness at each follow-up. Following the recommendation of Shortreed et al. (2014) [201], we will employ conditional imputation models for missing data that take advantage of the time- and stage-ordered nature of the trial design. At each time point, missing data (outcomes and covariates) will be imputed using baseline, outcome measures, randomized treatments, and remission indicators measured prior to the time of the missing data collection. For participants who are lost to follow-up prior to the Stage 2 randomization, we will perform a single imputation, assigning a status of non-remitter (i.e., non-responder) at 12 weeks. Multiply imputed datasets will then include an imputation for the missing Stage 2 randomization and imputations of subsequent outcomes. We will document and appropriately follow participants depending on their type of dropout (e.g., dropout from study only but maintaining physician/site service, vs dropout from study and service). Dropouts and reasons will be summarized in the final trial CONSORT diagram. Sensitivity analyses will compare results (treatment means, treatment group differences, and statistical conclusions regarding group differences) from models with only complete cases with results from models incorporating missing data through multiple imputation.

### Heterogeneity of treatment effects (HTE)

Our study data will include a rich body of information on *tailoring variables*, including baseline individual, family, and context characteristics, some related directly to child anxiety and its clinical portrait: past treatment response, family history of anxiety, SES, and variables that are *potentially modifiable*, such as overall symptom severity [29, 31], functional impairment [29, 31], severity of depression and other comorbid illnesses, treatment fidelity and adherence, medication dose, treatment setting (community or university; primary pediatric or specialty mental health clinic), and parental depression [111] or anxiety [112, 202]. These tailoring variables, used in post-hoc analyses, will help shed light on how patterns of response through the various pathways of this SMART study may relate to individual, family, or context characteristics at the beginning of treatment. One important product from this trial will be the development of a prospective, adaptive intervention algorithm – a set of tailored clinical pathways based on (a) participant characteristics at baseline and (b) response to intervention after acute intervention. This adaptive treatment algorithm will have immediate clinical applicability in populations of diverse and vulnerable youth.

#### HTE analysis goals: pre-specified hypotheses and supporting evidence base

Based on findings from prior studies, we hypothesize that predictors of poor acute treatment outcomes will include lower SES [31, 32], ethnic minority status [30, 41], comorbid depression [203], a diagnosis of social anxiety disorder [28, 31, 32, 204], and treatment in a pediatric rather than a specialty mental health clinic [31]. More severe anxiety will predict better response to Stage 2 combined CBT + fluoxetine treatment than either treatment modality alone [49, 50].

#### HTE analysis plan

Dividing the total sample into pre-defined subgroups, we will use analytic methods detailed above to estimate treatment effects across subgroups. Forest plots of mean treatment group differences with confidence intervals for each subgroup will be completed to graphically evaluate uniformity of treatment effect. In the total sample, we will first add the subgroup as a main effect covariate. Evaluation of the subgroup main effect will test over the entire sample (combined intervention groups) whether and to what extent outcomes in general differs by SES, ethnicity, comorbid depression, social anxiety disorder, and treatment setting.

We will employ Q-learning as our primary approach to testing the heterogeneity of treatment effects. This is a regression approach recommended for SMART data to identify tailoring variables that modify treatment responses and suggest enhancements to the sequential decision-making of an adaptive intervention [197]. In this 2-stage SMART design with a continuous trial outcome, Q-learning with linear regression will be used in 2 steps. In step 1, the Stage 2 decision rule will be optimized among Stage 1 non-remitters, identifying individual variables that significantly modify the Stage 2 randomization effect. In step 2, the Stage 1 decision rule will be optimized, controlling for the optimized Stage 2 intervention and identifying individual variables that significantly modify the Stage 1 randomization effect. This approach therefore may suggest a more tailored adaptive intervention that could be evaluated in a future SMART study.

As there will remain clinical subgroups of primary interest to clinicians, we will also assess in a secondary analysis the randomized treatment-by-subgroup (e.g., treatment-by-SES group) interaction terms to estimate and test for differences in treatment effects (Main Effects 1 & 2) by subgroup. Estimating treatment effects across our carefully pre-defined subgroups, we will use forest plots of mean treatment group differences with confidence intervals for each subgroup to show subgroup-specific effects and graphically evaluate uniformity of treatment effects.

With an estimated 20% dropout, we will be able to detect the following effect sizes for Main Effect 1 (total *n* = 324) in various subgroups by sample representation (n): (1) Effect Size = 0.40 for 60% representation (*n* = 194); (2) Effect Size = 0.44 for 50% representation (*n* = 162); (3) Effect Size = 0.50 for 40% representation (*n* = 130). For Main Effect 2 (total n = 194), we will be able to detect: (1) Effect Size = 0.52 for 60% representation (*n* = 116); (2) Effect Size = 0.58 for 50% representation (*n* = 96); (3) Effect Size = 0.64 for 40% representation (*n* = 78). Subgroup interactions will be tested formally; with the total anticipated sample size of 324 to complete the 24-week intervention, we will have 80% power to detect subgroup differences in treatment effect sizes of ~ 0.65 and higher for Main Effect 1, and ~ 0.8 and higher for Main Effect 2.

In additional exploratory analyses, we will use latent class/profile analysis to model heterogeneity of outcome response patterns, assuming categorical latent variables (latent groups) for response. Latent class/profile analysis has been proposed as an alternative to subgroup analysis in clinical trials. As an exploratory analysis, it uses observed data (e.g., clinical characteristics, randomized treatments) to identify latent group classifications that may suggest individual characteristics related to greater responses to treatment [205].

In addition to assessing the effects of telehealth delivery of treatment on outcomes, we will assess whether the percentage of treatment sessions conducted via telehealth moderates treatment outcomes. This test will tell us whether discontinuities in telemedicine use over the course of the study or within individual patients has influenced our findings. We will also assess whether session attendance and trial completion differ by degree of participation in telehealth vs in-person intervention.

#### Plan to report pre-specified analyses

In our primary outcome paper, we will include results of our pre-specified subgroup analyses. We will name and report all pre-specified subgroups and their rationale for inclusion, the number of post-hoc HTE analyses, and outcomes analyzed. Reporting will include graphical forest plots, with estimated treatment group differences and confidence intervals for all subgroups, as well as tests of treatment-subgroup interactions.

### Mediation analyses

**Fig. 6:**
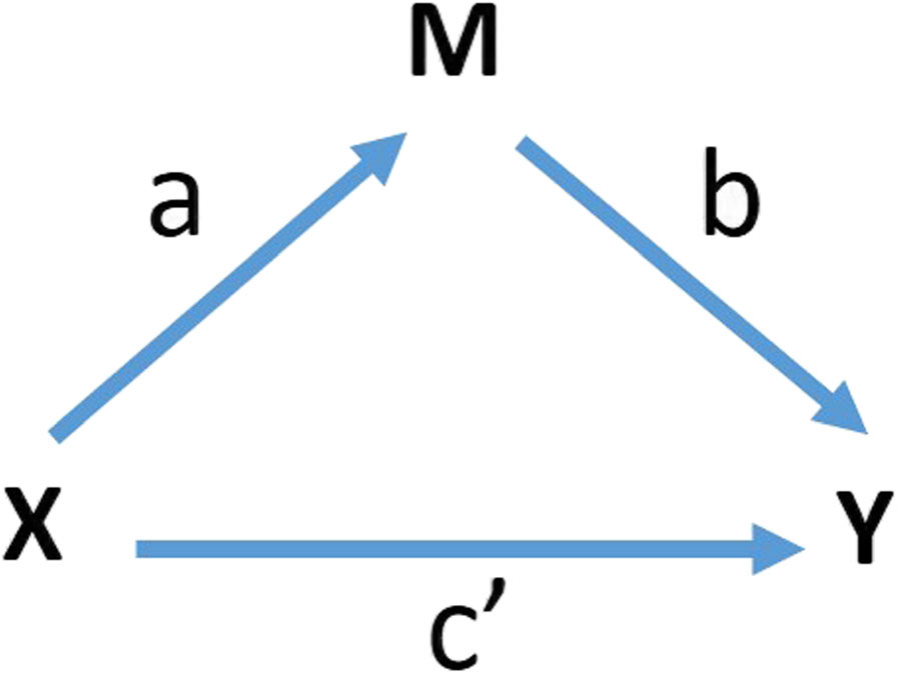
Tests of Mediation. X = Treatment. M = Proposed Mediator. Y = 24-week outcomes. a = coefficient for correlation of X with M (M = aX). b = coefficient for correlation of M with Y (Y = bM). Y = c X = c’ + ab (the total effect of X on Y). M = a X (the effect of X on M). Y = c’ X + b M (direct effect of X on Y). a x b (mediation effect). Algebraic sign of a x b x c (type of mediation – complementary or suppressive)

We will perform mediation analyses on the Stage 1 main intervention effects and will follow the literature for development of statistical approaches for mediation analysis of SMART design adaptive interventions. We will test whether the common and specific factors in our causal pathway model significantly mediate the associations of treatment with patient outcomes. Let X be the assigned treatment, Y be 24-week outcomes, and M be the proposed mediator **(**Fig. 6**)**. We will assess mediation using 3 regression equations: 1) *Y* = *c*_1_*X* + *e*_1_, assessing the association of treatment with 24-week outcomes; 2) *M* = *aX* + *e*_2_, assessing the association of treatment with the putative mediator; and 3) *Y* = *c*_2_*X* + *bM* + *e*_3_, associating the association of treatment with 24-week outcome when adjusting for the mediator (termed the “direct effect” of X with Y). Age and sex will be included as covariates in all three equations. We will test whether the estimator of the indirect effect (*a x b*) differs significantly from zero using bias-corrected bootstrapped confidence intervals on the estimator. A significant mediating effect suggests that the association of treatment effects (X) with trial outcomes (Y) in regression (1) is in part explained by treatment effects on mediating outcomes (M) in regression (3).

We then will assess whether mediation is partial or complete, and whether it is complementary (i.e., the direct and mediated effects are in the same direction on the outcome Y and therefore have the same algebraic sign) or suppressive (the direct and mediated effects are in opposing directions on outcome Y and therefore have opposite signs). We will assess the significance of the direct effect (coefficient c_2_ in eq. 3): statistical significance of this term will signify that mediation by M is partial [206, 207]. We will also assess the algebraic sign of the product a*b*c to determine whether mediation is complementary or suppressive [206, 207].

Finally, a complex mediation model that jointly estimates multiple mediating outcomes and incorporates the correlations among mediators and joint effects of mediators on trial outcomes will ultimately be used to test and understand the mechanisms of the CBT and medication interventions on anxiety outcomes. Moderator effects of the Contextualizing Factors will be tested as described under “Heterogeneity of Treatment Effects”.

### Data safety monitoring board (DSMB)

Our data safety-monitoring plan is designed to ensure the safety of participants, the validity of the data collected, and the appropriate termination of the study in the event that significant benefits or risks are uncovered, or if it appears that the trial cannot be concluded successfully. We will convene a Data Safety Monitoring Board (DSMB) that is fully independent from the sponsor and competing interests. The DSMB will have the authority to recommend termination of the trial to the principal investigator and funding agency if it judges that a specific action is not in the best interest of study participants or that the conduct of study processes are unlikely to lead to sound scientific results. The board will also review subject burden levels associated with data collection tasks. The board will meet every six months to review study progress and adverse events. The PI and study staff will provide to the DSMB all patient data collection materials as well as a semi-annual summary report on patient outcome tracking. They also will immediately report to the DSMB any adverse patient or caregiver outcomes. The DSMB will be authorized to request any additional information or study materials it deems appropriate. All participants will be provided contact information for the DSMB to register complaints or other problems.

The DSMB will comprise three health care researchers, who are voting members, and one non-voting caregiver. They are:

- Daniel Pine MD, Chief, Section on Development and Affective Neuroscience in the National Institute of Mental Health Intramural Research Program. He is an expert in the neurobiology and treatment of pediatric anxiety and mood disorders. He is chair of the DSMB.
- Armando Andres Piña, Ph.D., Associate Professor in the Department of Psychology at Arizona State University. He is an expert in real-world psychosocial interventions for pediatric anxiety disorders.
- Ravinder Anand, Ph.D. Vice President and Biostatistician, The Emmes Company, LLC, Rockville, MD. He is an expert clinical trials statistician. He is the clinical trials statistician for the Pediatric Trials Network.
- Christine Norene Smith: non-voting community stakeholder

The charter for the DSMB is available from either the study PI or the DSMB chair upon request. No independent audits for the study are planned.

### Ethics

This study protocol was reviewed and approved under the SMART Institutional Review Board (IRB) mechanism, with Children’s Hospital Los Angeles the designated lead IRB. Parents will provide informed written consent for their child’s participation, and the child will provide informed written assent (see Supplementary Material for the consent and assent forms for the clinical trial and ancillary study), which will be obtained by trained study personnel. Post-trial care will be determined through routine clinical decision-making of the patient and treating clinician, and it will be paid through the patient’s usual insurance mechanisms. No compensation will be provided for those who suffer harm from trial participation. Any changes to the study protocol will need to be reviewed and approved by PCORI and the IRB. Changes will be documented within ClinicalTrials.gov and communicated to all relevant parties (e.g., investigators, DSMB, and trial participants).

#### Protection of privacy

We will minimize risks for confidentiality breaches in several ways. Research staff members who collect study data will do so only from one of our 9 clinical facilities or research sites. Study personnel will be required to sign confidentiality agreements and will be trained in the protection of human subjects. No data containing participant identifiers will be transferred outside CHLA or USC. In lieu of participant names or medical record numbers, participants will be identified only by a random subject ID number on both the raw and electronic study data. Crosswalks that link a participant’s name and medical record number to that person’s study number will be kept by the Principal Investigator in a locked file and will be destroyed at the end of the study. Medical service use data and participant names and contact information will be kept on a secured, non-networked computer (i.e., it will have no Internet access), which will be stored in a locked, secured office. The computer will be password-protected; only the study PI and system analyst will be permitted to access it.

## Discussion

### Dissemination and implementation (DI)

The overarching aim of our efforts will be to disseminate and promote the appropriate uptake of our research findings and to facilitate the use of high-quality, relevant evidence by patients, caregivers, clinicians, insurers, and policy-makers in reaching better-informed decisions. The following provides examples of our DI work, which will be intensive and intentional, involving the active process of identifying target audiences and tailoring communication strategies to both a) increase awareness and understanding of the research findings, and b) motivate their appropriate use in policy, practice, and informing individual patient choices.

#### Project-specific DI repository and website

Our website will host: a) study materials and protocols (e.g., recruitment, retention, treatment, assessment), b) newsletters for study participants, participating sites, collaborating agencies and other interested stakeholders, c)Meeting agendas and minutes from the Parent and Youth Leadership Advisory Committees, d) study findings (full peer-reviewed reports plus brief summaries), e) lessons learned while conducting the research, f) brief videos and testimonials from investigators and Advisory Council members, and g) other DI products. This website will also serve as a clearinghouse for information for our study participants, who will also receive frequent study updates in English and Spanish.

#### Peer-reviewed journals and professional conferences

Study methods, findings, and supporting information will be published in highly-visible peer-reviewed journals, augmented by presentations at key professional conferences. Our Advisory Council members will be invited to co-author manuscripts and co-present.

#### Partnering with other key stakeholders

We will partner with other key organizations to disseminate study findings, such as the *Anxiety and Depression Association of America* (ADAA), which serves as both a professional membership organization and an organization that provides evidence-based information to the public; last year the ADAA had over 38 million visitors to its website. Others include *NIMH Outreach Partners*, a nationwide program charged with disseminating research findings and educational materials to the public, including to populations that experience mental health disparities. Venues will include weekly social media updates (#MentalHealthMondays) and monthly newsletters. These will link to the study findings and final report posted on the PCORI website per standard PCORI practices.

Multimedia Presentations and Displays These can be effective methods for sharing research results. We will build on existing relationships and use novel and innovative approaches to DI. For example, we will partner with *Hollywood Health and Society* (HHS), a program of USC’s Annenberg School of Communication that provides entertainment professionals with accurate and timely information for storylines on health. The SC CTSI currently works with HHS and television writers and producers to include storylines about clinical trials participation, which has resulted in an award-winning storyline on *Grey’s Anatomy* and storylines for *Life Sentence*, *The Fosters,* and *Empire*. With HHS, the SC CTSI also partnered with Life Noggin to develop a brief cartoon video that describes the importance of clinical trial participation (https://youtu.be/BYZusIKpHIA); the YouTube video received over 150,000 views in the first two days. We will use these same approaches to disseminate information about pediatric anxiety, the importance of evidence-based treatments, and study findings. In addition, we will partner with WeRise LA, which uses art to encourage youth to foster the empowerment of mental health and wellness. WeRise LA hosts an annual youth-driven art exhibit that encourages dialogue to reduce stigma surrounding mental health treatments.

#### Our community/our health Los Angeles (OC/OH-LA)

This is an approach we have used previously to facilitate a dialogue between researchers and members of the lay community about science and the implications of scientific findings. In addition, a consortium of CTSAs across the country have worked together to coordinate these events using simulcast technology. We will conduct at least one annual local and/or national OC/OH event that focuses on this research.

#### Uptake and adoption of evidence-based findings

We will draw upon the DI expertise of our team, collaborating partners, and stakeholder advisors to ensure early engagement and ongoing relevance of our research through continuous ties to key stakeholder groups likely to be interested and available to support our DI efforts. For example, the depression care initiative of our partner site, Kaiser Permanente Southern California (KPSC), has selected several high priority expansion populations, including adolescents with anxiety. Thus, the organization has already committed to creating an anxiety treatment program for adolescents 12 years and older. Our study offers a perfect opportunity to provide needed training, ongoing technical assistance, and oversight for the uptake and adoption of adolescent anxiety treatment, at KPSC and throughout the Kaiser healthcare system. We are confident that the program established with this study will be institutionalized within KPSC if findings warrant this and can be adapted to other local healthcare systems including AltaMed, Los Angeles County, and DHS.

#### Authorship eligibility guidelines

We will follow the guidelines of the International Committee of Medical Journal Editors for authorship eligibility [208]. All 4 of the following criteria must be met to be considered an author: (1) Substantial contributions to the conception or design of the work; or the acquisition, analysis, or interpretation of data for the work; (2) Drafting the work or revising it critically for important intellectual content; (3) Final approval of the version to be published; (4) Agreement to be accountable for all aspects of the work in ensuring that questions related to the accuracy or integrity of any part of the work are appropriately investigated and resolved. All contributors who do not meet these 4 criteria for authorship will be listed in the ‘Acknowledgements’ section of the paper.

## Data Availability

Full public access of the entire de-identified, participant-level dataset and statistical code will be made available 1 year after the report of primary outcomes is published.

## Abbreviations

ADHD: Attention-Deficit/Hyperactivity Disorder
ADIS: Anxiety and Related Disorders Interview Schedule
AE: Adverse event
AHRQ: Agency for Healthcare Research and Quality
CAIS: Child Anxiety Impact Scale
CAMS: Child/Adolescent Anxiety Multimodal Study
CBCL: Child Behavioral Checklist
CBT: Cognitive behavioral therapy
CHLA: Children’s Hospital Los Angeles
C-SSRS: Columbia-Suicide Severity Rating Scale
DSM: Diagnostic and Statistical Manual
DSMB: Data Safety Monitoring Board
ES: Effect size
FDA: Food and Drug Administration
HTE: Heterogeneity of Treatment Effects
iCAN: International Children’s Advisory Network
IRB: Institutional Review Board
KSADS-COMP: Schedule for Affective Disorders and Schizophrenia for School-Aged Children, Computerized version
LAC: Los Angeles County
OCD: Obsessive-Compulsive Disorder
OC/OH-LA: Our Community/Our Health Los Angeles
PI: Principal investigator
PLC: Parent Leadership Council
PTSD: Post-Traumatic Stress Disorder
RCT: Randomized controlled trial
REDCap: Research Electronic Data Capture
ROC: Receiver Operator Characteristics
SCARED: Screen for Child Anxiety Related Disorders
SC CTSI: Southern California Clinical and Translational Science Institute
SD: Standard deviation
SES: Socioeconomic status
SMART: Sequential Multiple Assignment Randomized Trial
SNRI: Serotonin and norepinephrine reuptake inhibitor
SSRI: Selective serotonin reuptake inhibitor
TAU: Treatment As Usual
UCEDD: University Centers of Excellence in Developmental Disabilities
YLC: Youth Leadership Council
